# Supplementary material for: G‐Quadruplex Recognition by DARPIns through Epitope/Paratope Analogy
Source: Chemistry. 2022 Aug 22;28(57):e202201824. doi: 10.1002/chem.202201824 (PMC9804223; doi:10.1002/chem.202201824)
Supplement: Supplementary file 1 — Supporting Information [file CHEM-28-0-s001.pdf]

Chemistry–A European Journal

Supporting Information

G-Quadruplex Recognition by DARPIns through Epitope/Paratope Analogy

Tom Miclot,* Emmanuelle Bignon, Alessio Terenzi, Stéphanie Grandemange, Giampaolo Barone,* and Antonio Monari*

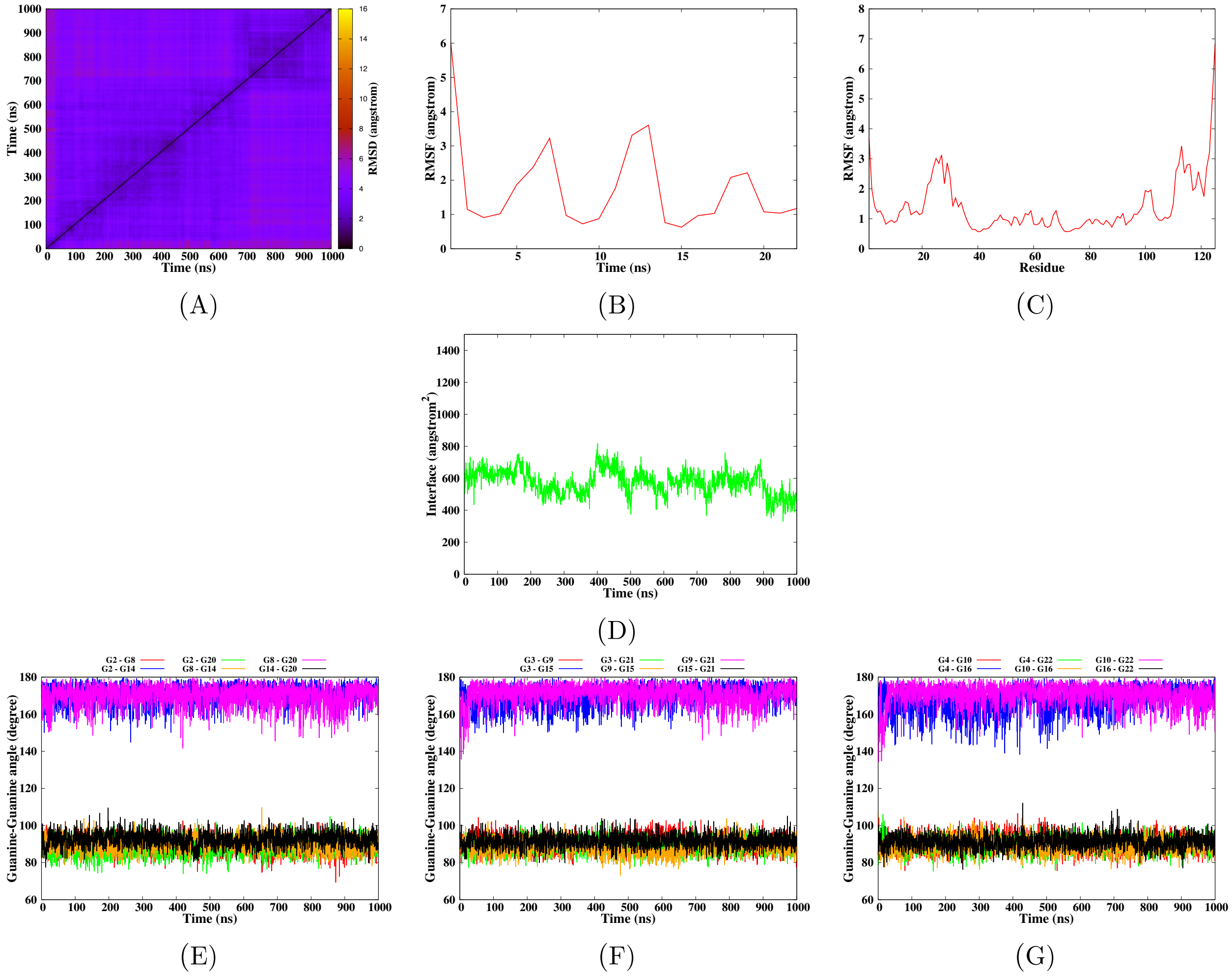

Figure S1 – Simulation of the h-Telo G-quadruplex DNA in interaction with 2E4 according to the model 1-1, run 1. The convergence of the simulation is given by the RMSD-2D map of the DNA-Protein complex (A). The mobility of the DNA and protein residues is given by their root mean square fluctuation (B-C). Surface of the interaction interface between the protein and G-quadruplex (D). Finally the structural parameters of the G-quadruplex are given by the angles between the guanines for each tetrad (E-G).

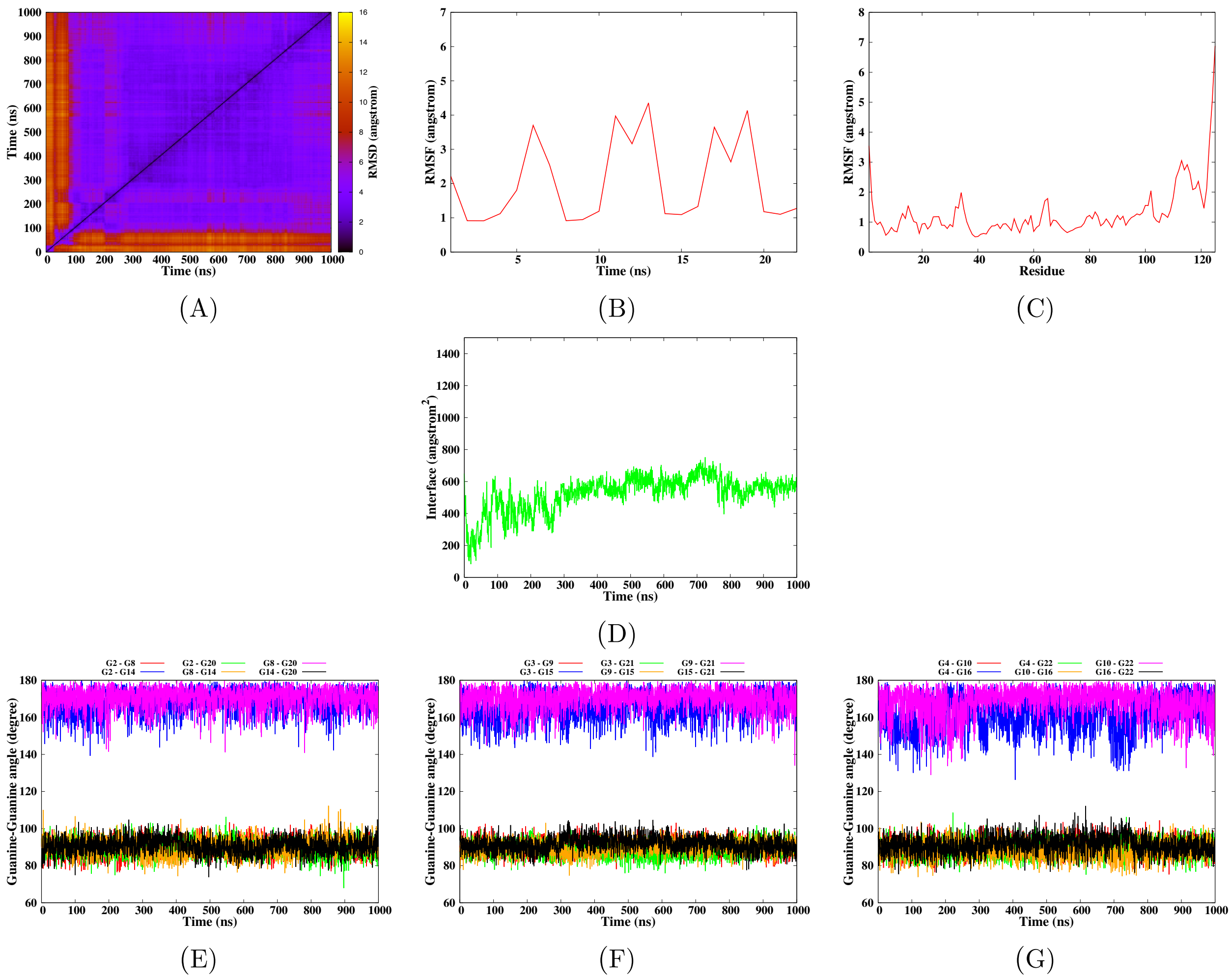

Figure S2 – Simulation of the h-Telo G-quadruplex DNA in interaction with 2E4 according to the model 1-1, run 2. The convergence of the simulation is given by the RMSD-2D map of the DNA-Protein complex (A). The mobility of the DNA and protein residues is given by their root mean square fluctuation (B-C). Surface of the interaction interface between the protein and G-quadruplex (D). Finally the structural parameters of the G-quadruplex are given by the angles between the guanines for each tetrad (E-G).

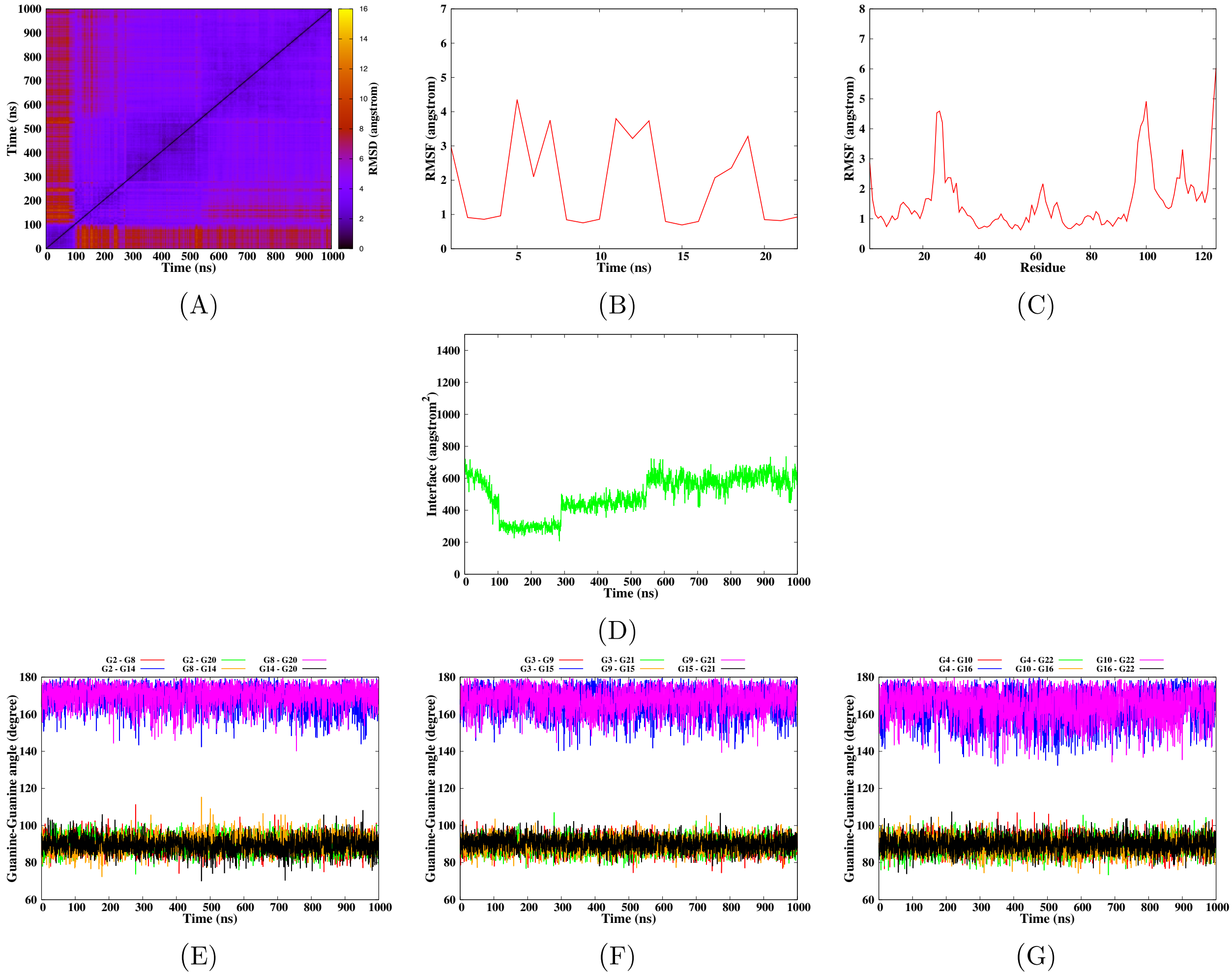

Figure S3 – Simulation of the h-Telo G-quadruplex DNA in interaction with 2E4 according to the model 6-1, run 1. The convergence of the simulation is given by the RMSD-2D map of the DNA-Protein complex (A). The mobility of the DNA and protein residues is given by their root mean square fluctuation (B-C). Surface of the interaction interface between the protein and G-quadruplex (D). Finally the structural parameters of the G-quadruplex are given by the angles between the guanines for each tetrad (E-G).

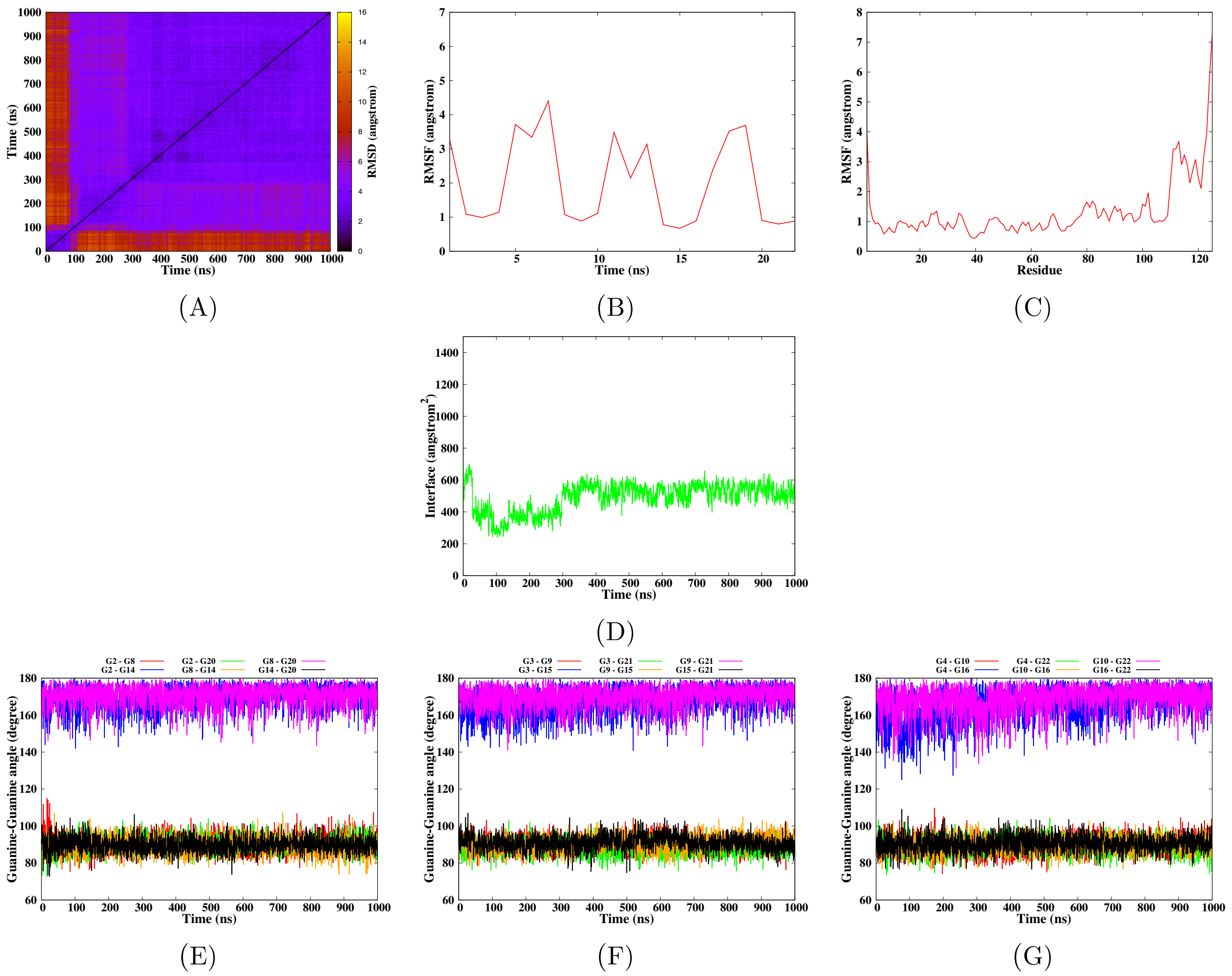

Figure S4 – Simulation of the h-Telo G-quadruplex DNA in interaction with 2E4 according to the model 6-1, run 2. The convergence of the simulation is given by the RMSD-2D map of the DNA-Protein complex (A). The mobility of the DNA and protein residues is given by their root mean square fluctuation (B-C). Surface of the interaction interface between the protein and G-quadruplex (D). Finally the structural parameters of the G-quadruplex are given by the angles between the guanines for each tetrad (E-G).

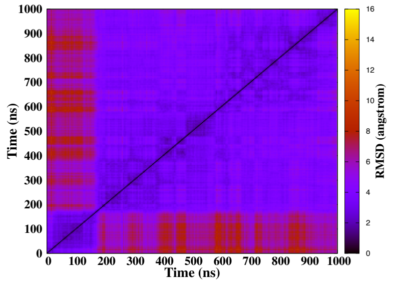

(A)

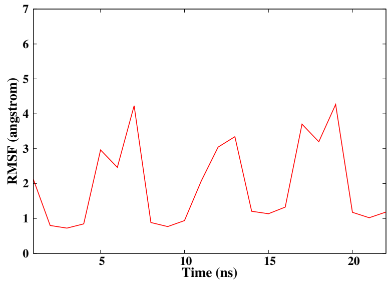

(B)

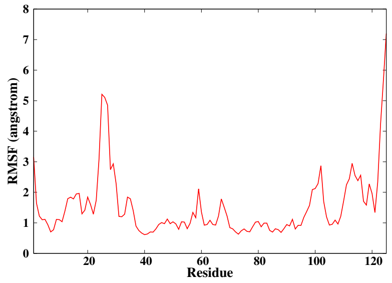

(C)

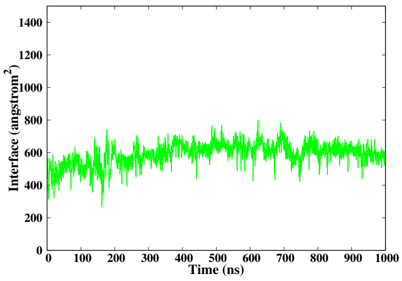

(D)

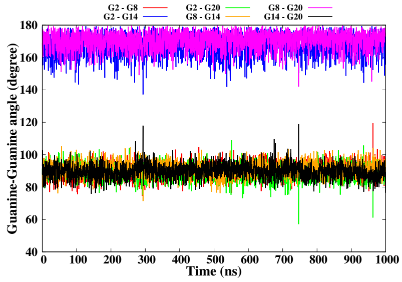

(E)

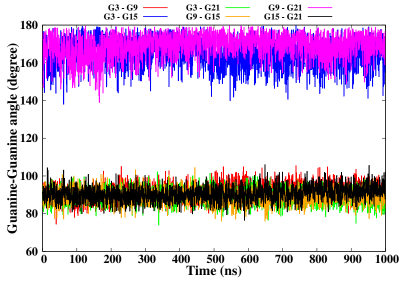

(F)

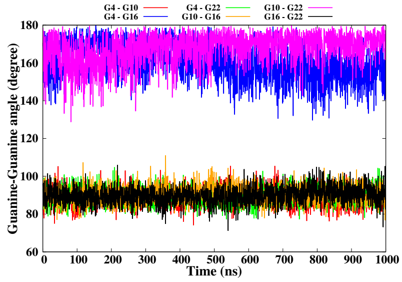

(G)

Figure S5 – Simulation of the h-Telo G-quadruplex DNA in interaction with 2E4 according to the model 8-4, run 1. The convergence of the simulation is given by the RMSD-2D map of the DNA-Protein complex (A). The mobility of the DNA and protein residues is given by their root mean square fluctuation (B-C). Surface of the interaction interface between the protein and G-quadruplex (D). Finally the structural parameters of the G-quadruplex are given by the angles between the guanines for each tetrad (E-G).

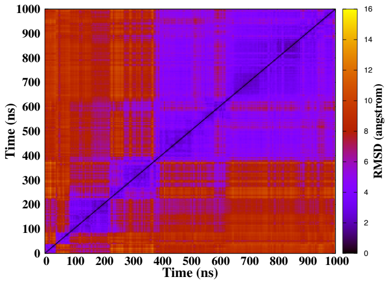

(A)

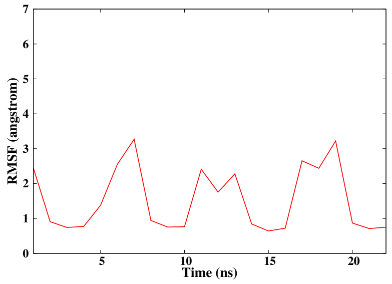

(B)

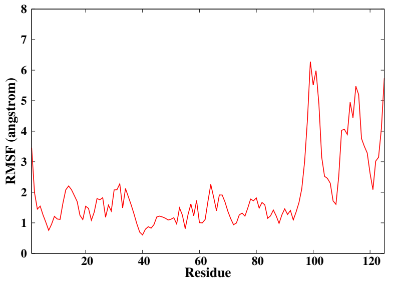

(C)

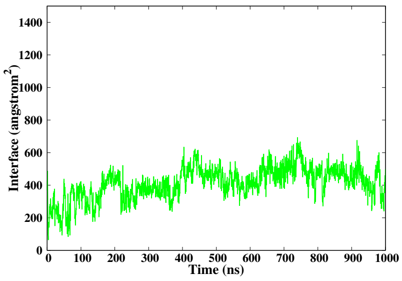

(D)

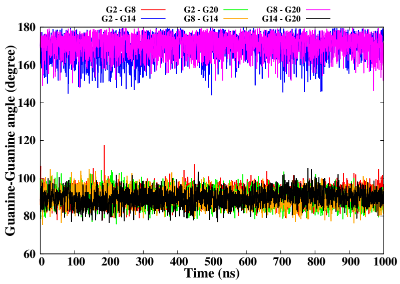

(E)

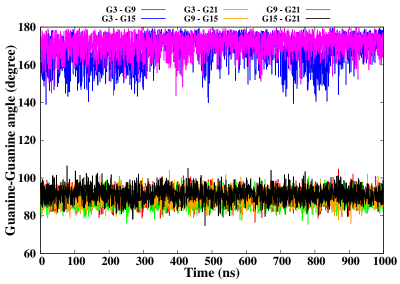

(F)

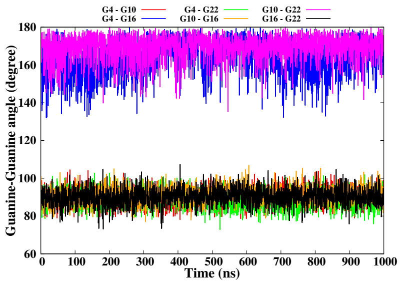

(G)

Figure S6 – Simulation of the h-Telo G-quadruplex DNA in interaction with 2E4 according to the model 8-4, run 2. The convergence of the simulation is given by the RMSD-2D map of the DNA-Protein complex (A). The mobility of the DNA and protein residues is given by their root mean square fluctuation (B-C). Surface of the interaction interface between the protein and G-quadruplex (D). Finally the structural parameters of the G-quadruplex are given by the angles between the guanines for each tetrad (E-G).

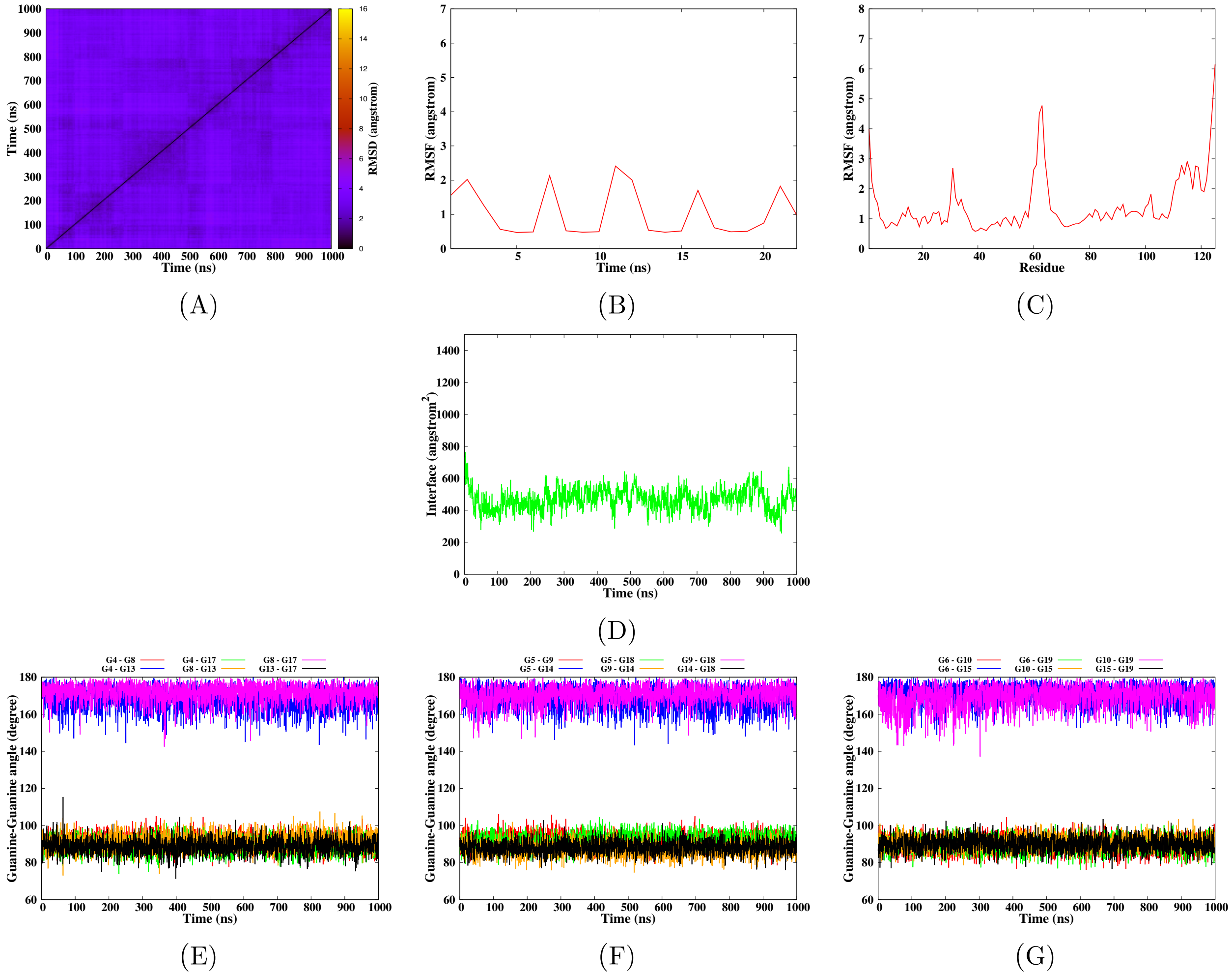

Figure S7 – Simulation of the c-Myc G-quadruplex DNA in interaction with 2E4 according to the model 1-1, run 1. The convergence of the simulation is given by the RMSD-2D map of the DNA-Protein complex (A). The mobility of the DNA and protein residues is given by their root mean square fluctuation (B-C). Surface of the interaction interface between the protein and G-quadruplex (D). Finally the structural parameters of the G-quadruplex are given by the angles between the guanines for each tetrad (E-G).

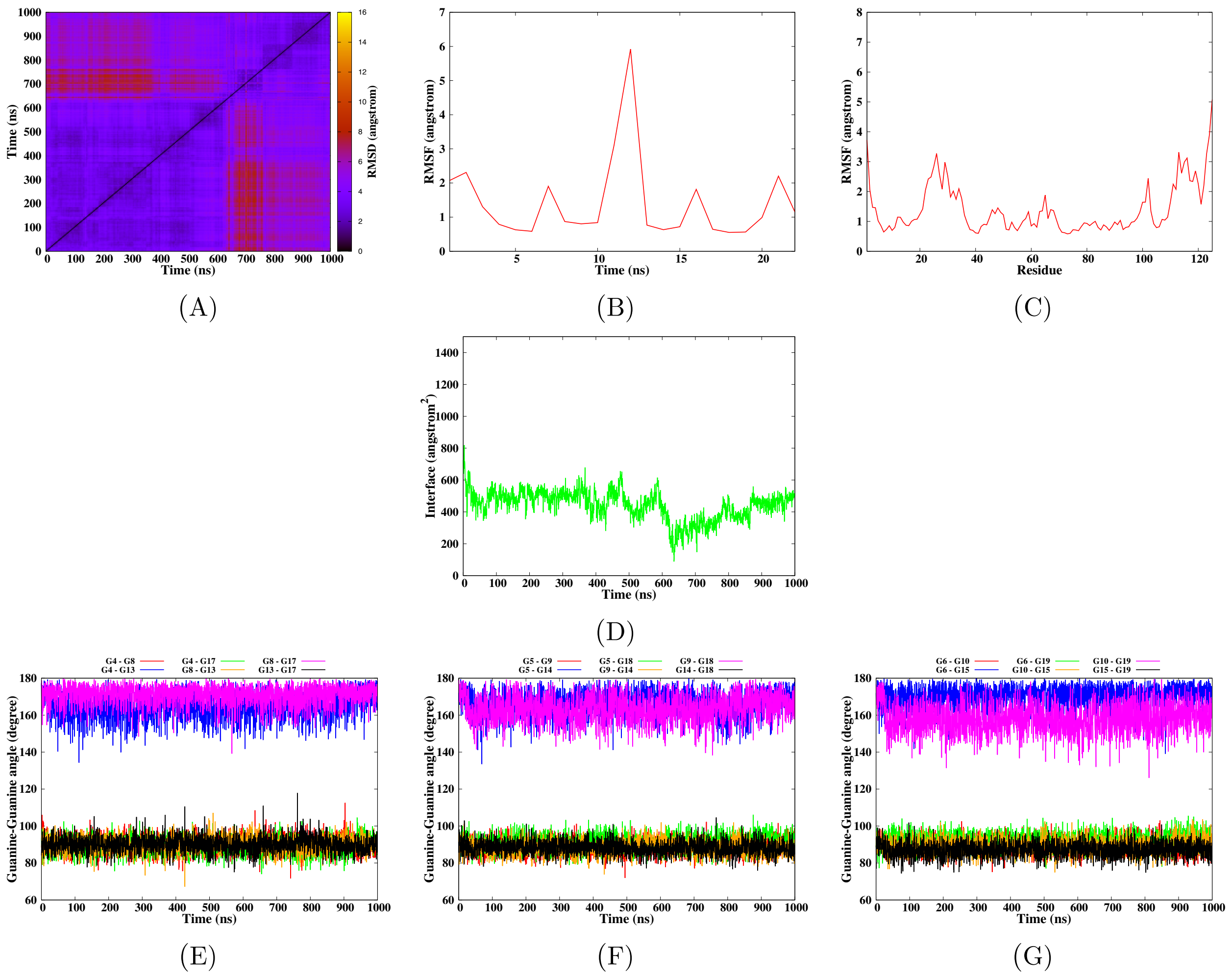

Figure S8 – Simulation of the c-Myc G-quadruplex DNA in interaction with 2E4 according to the model 1-1, run 2. The convergence of the simulation is given by the RMSD-2D map of the DNA-Protein complex (A). The mobility of the DNA and protein residues is given by their root mean square fluctuation (B-C). Surface of the interaction interface between the protein and G-quadruplex (D). Finally the structural parameters of the G-quadruplex are given by the angles between the guanines for each tetrad (E-G).

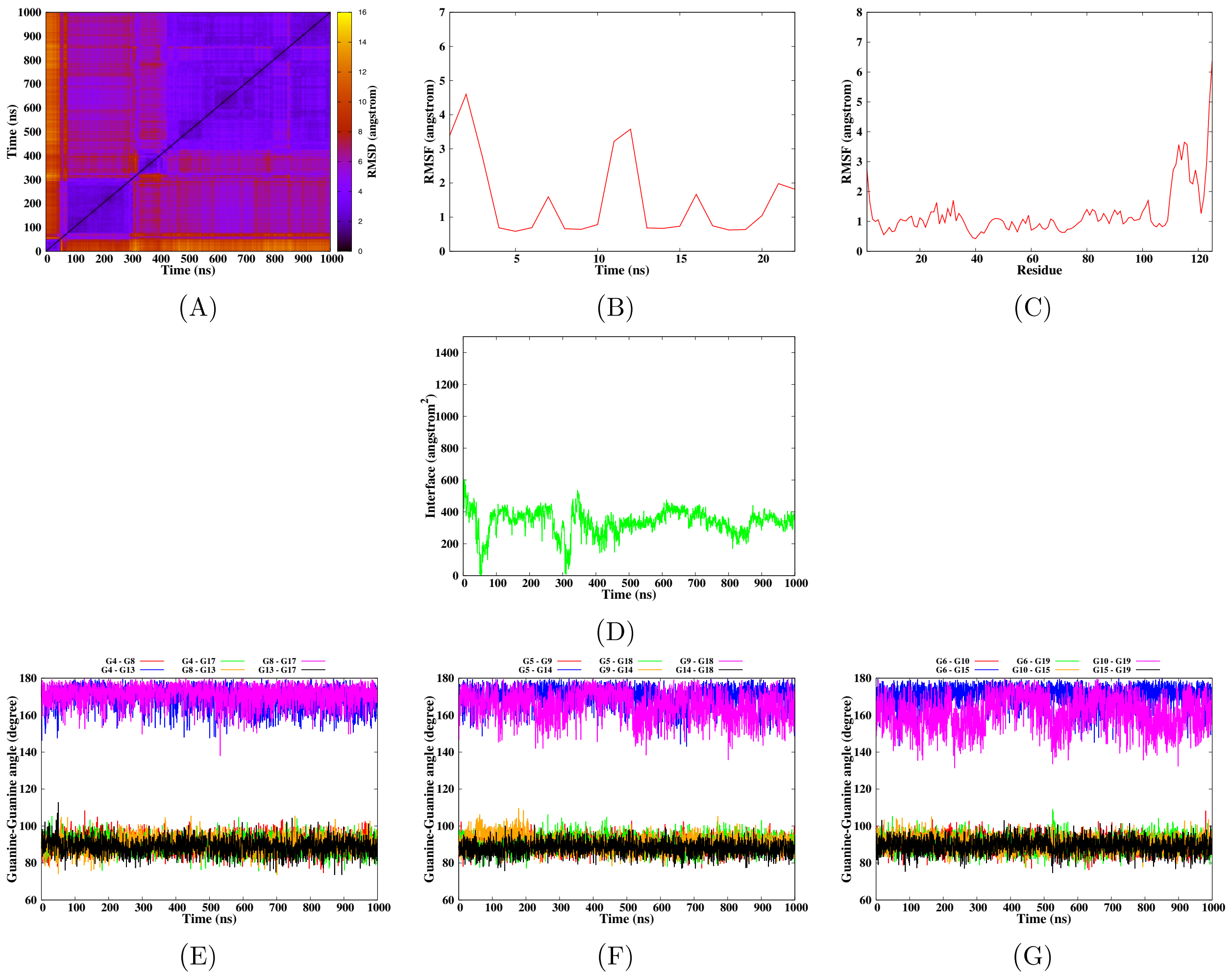

Figure S9 – Simulation of the c-Myc G-quadruplex DNA in interaction with 2E4 according to the model 5-4, run 1. The convergence of the simulation is given by the RMSD-2D map of the DNA-Protein complex (A). The mobility of the DNA and protein residues is given by their root mean square fluctuation (B-C). Surface of the interaction interface between the protein and G-quadruplex (D). Finally the structural parameters of the G-quadruplex are given by the angles between the guanines for each tetrad (E-G).

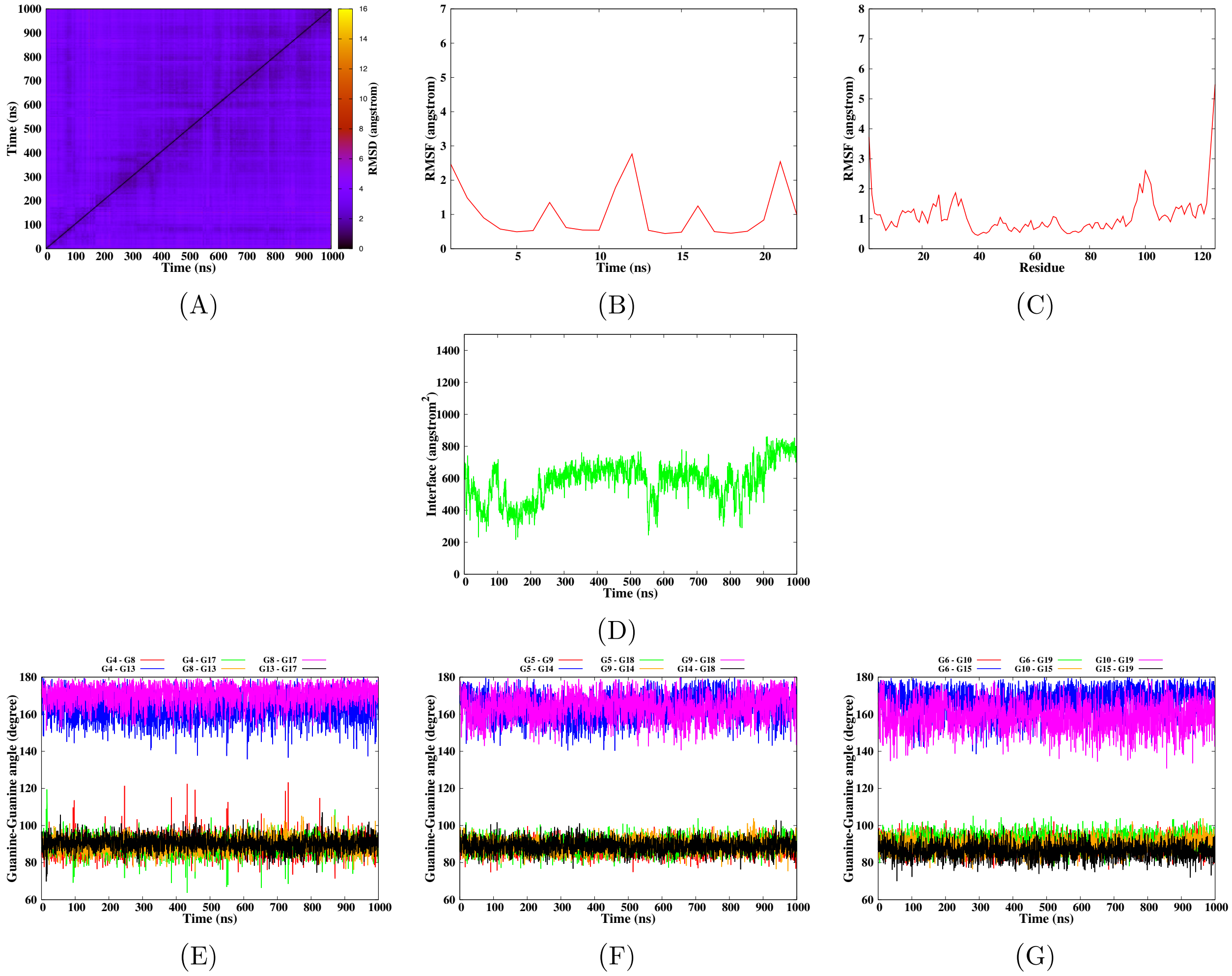

Figure S10 – Simulation of the c-Myc G-quadruplex DNA in interaction with 2E4 according to the model 5-4, run 2. The convergence of the simulation is given by the RMSD-2D map of the DNA-Protein complex (A). The mobility of the DNA and protein residues is given by their root mean square fluctuation (B-C). Surface of the interaction interface between the protein and G-quadruplex (D). Finally the structural parameters of the G-quadruplex are given by the angles between the guanines for each tetrad (E-G).

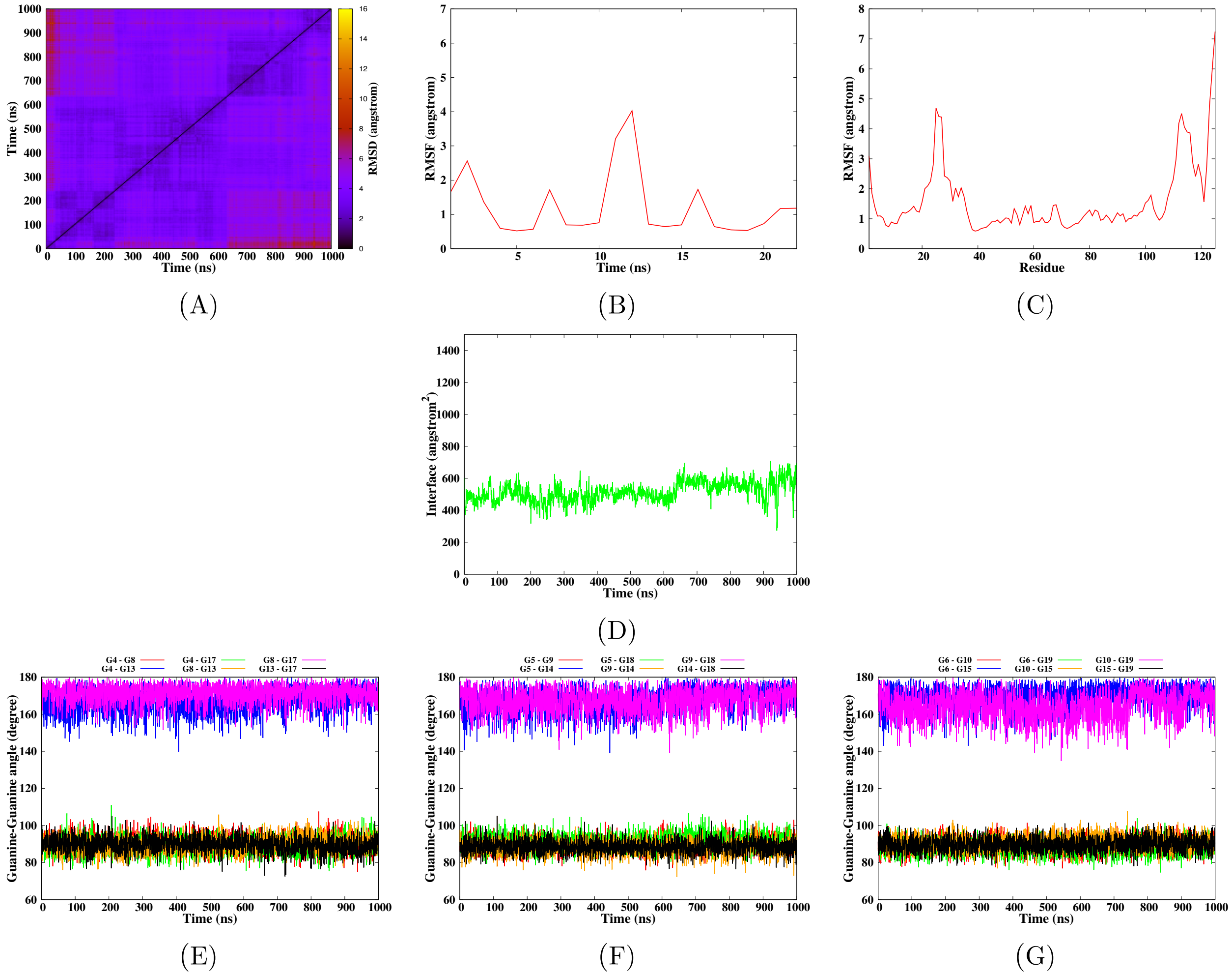

Figure S11 – Simulation of the c-Myc G-quadruplex DNA in interaction with 2E4 according to the model 6-4, run 1. The convergence of the simulation is given by the RMSD-2D map of the DNA-Protein complex (A). The mobility of the DNA and protein residues is given by their root mean square fluctuation (B-C). Surface of the interaction interface between the protein and G-quadruplex (D). Finally the structural parameters of the G-quadruplex are given by the angles between the guanines for each tetrad (E-G).

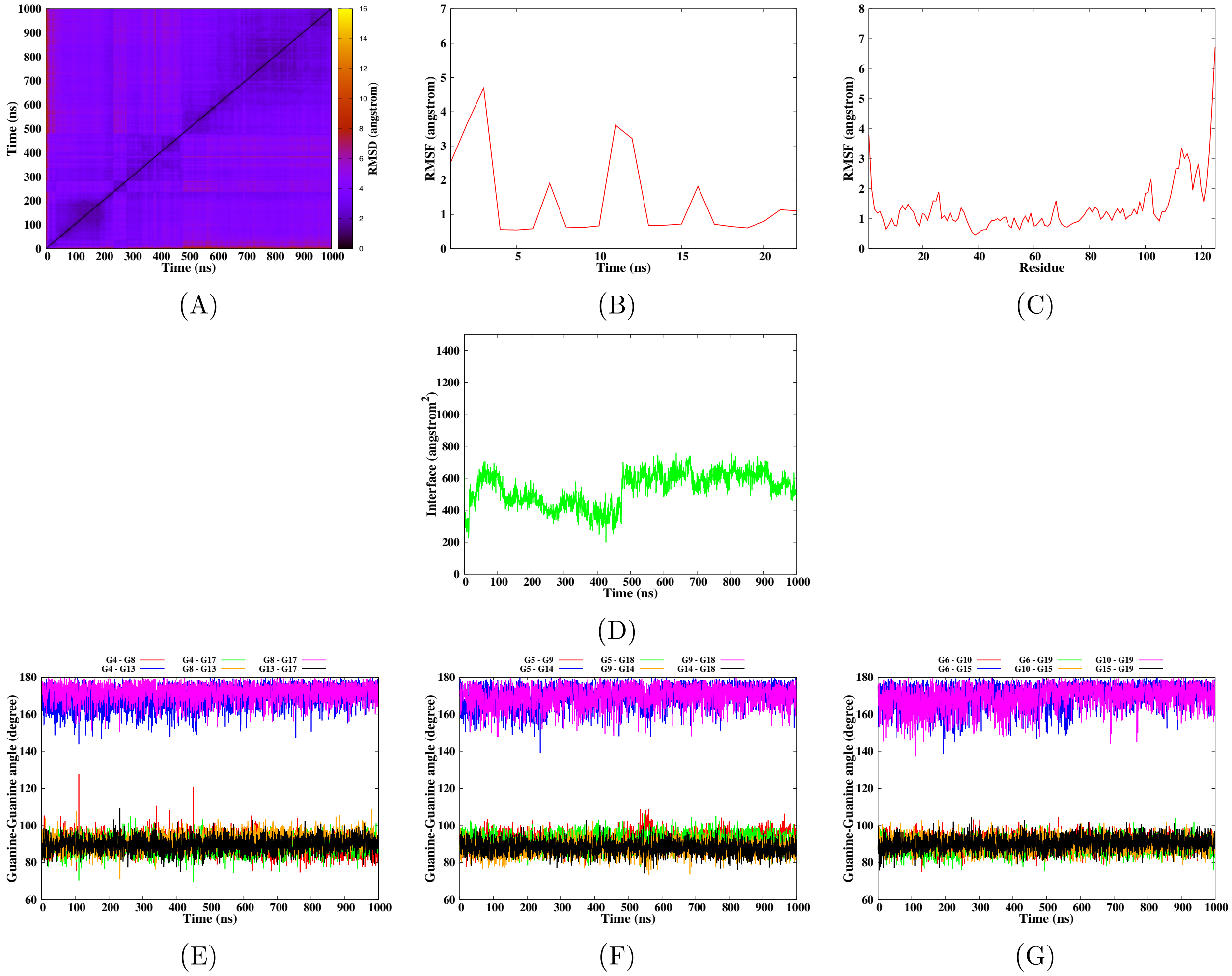

Figure S12 – Simulation of the c-Myc G-quadruplex DNA in interaction with 2E4 according to the model 6-4, run 2. The convergence of the simulation is given by the RMSD-2D map of the DNA-Protein complex (A). The mobility of the DNA and protein residues is given by their root mean square fluctuation (B-C). Surface of the interaction interface between the protein and G-quadruplex (D). Finally the structural parameters of the G-quadruplex are given by the angles between the guanines for each tetrad (E-G).

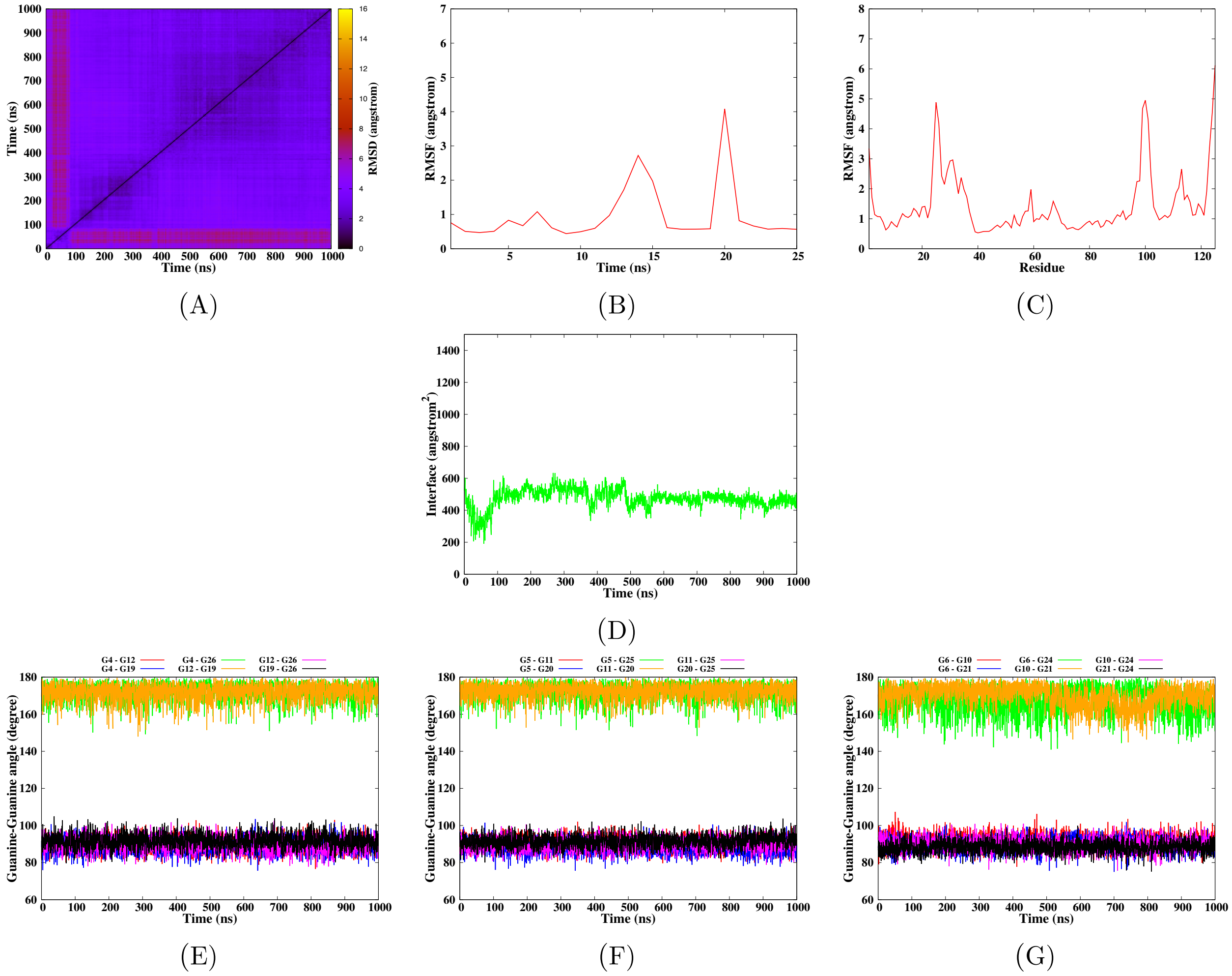

Figure S13 – Simulation of the Bcl-2 G-quadruplex DNA in interaction with 2E4 according to the model 1-1, run 1. The convergence of the simulation is given by the RMSD-2D map of the DNA-Protein complex (A). The mobility of the DNA and protein residues is given by their root mean square fluctuation (B-C). Surface of the interaction interface between the protein and G-quadruplex (D). Finally the structural parameters of the G-quadruplex are given by the angles between the guanines for each tetrad (E-G).

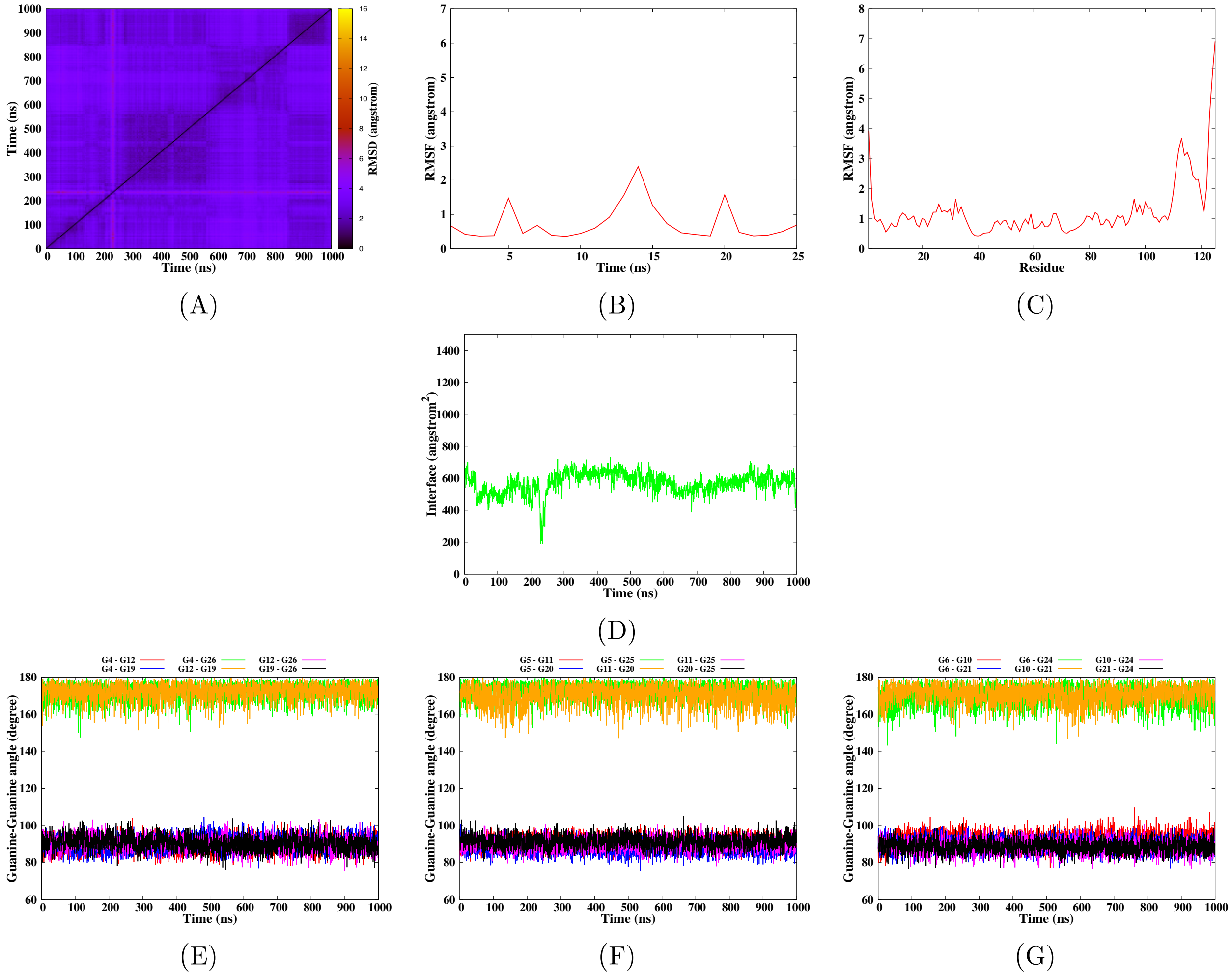

Figure S14 – Simulation of the Bcl-2 G-quadruplex DNA in interaction with 2E4 according to the model 1-1, run 2. The convergence of the simulation is given by the RMSD-2D map of the DNA-Protein complex (A). The mobility of the DNA and protein residues is given by their root mean square fluctuation (B-C). Surface of the interaction interface between the protein and G-quadruplex (D). Finally the structural parameters of the G-quadruplex are given by the angles between the guanines for each tetrad (E-G).

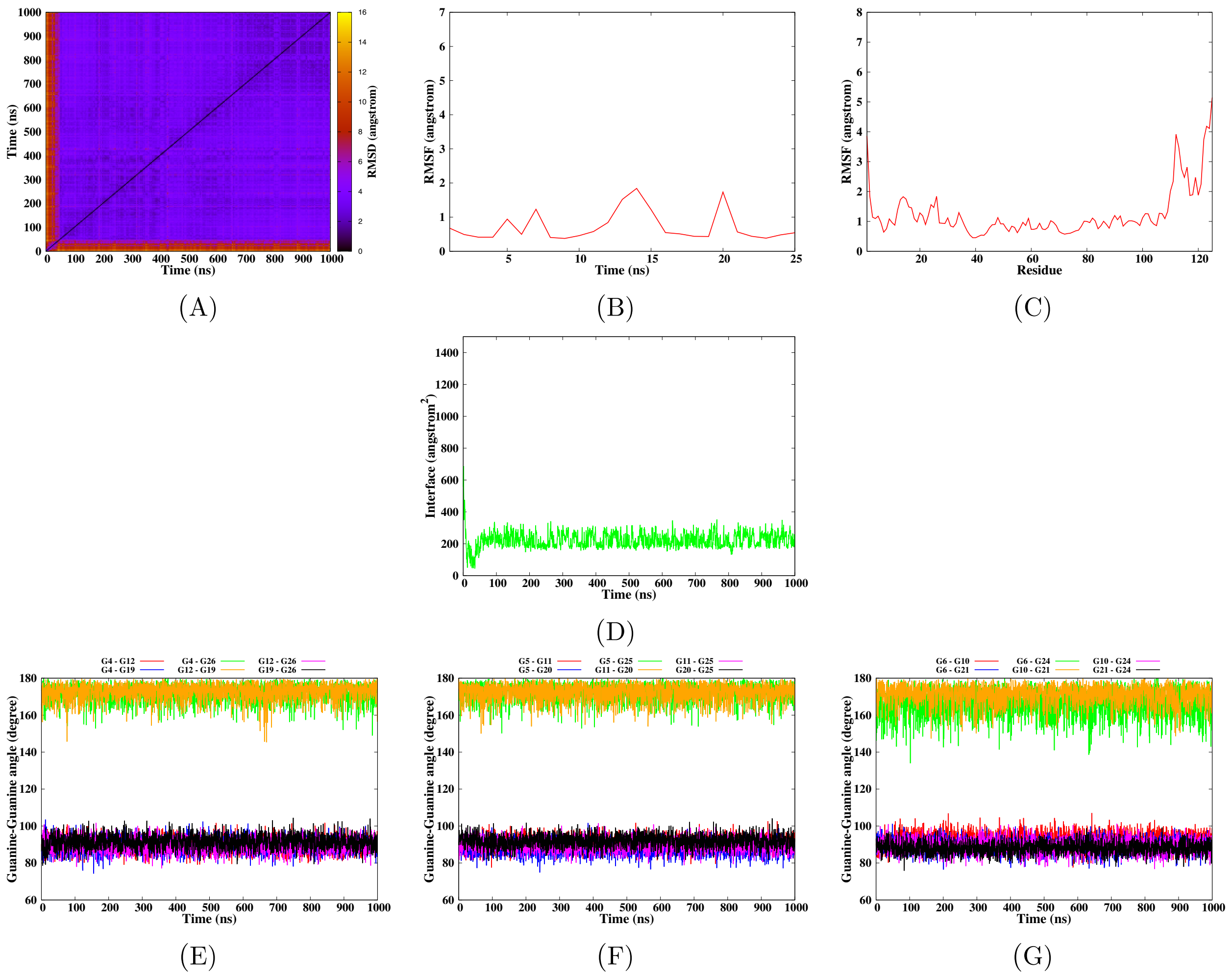

Figure S15 – Simulation of the Bcl-2 G-quadruplex DNA in interaction with 2E4 according to the model 5-1, run 1. The convergence of the simulation is given by the RMSD-2D map of the DNA-Protein complex (A). The mobility of the DNA and protein residues is given by their root mean square fluctuation (B-C). Surface of the interaction interface between the protein and G-quadruplex (D). Finally the structural parameters of the G-quadruplex are given by the angles between the guanines for each tetrad (E-G).

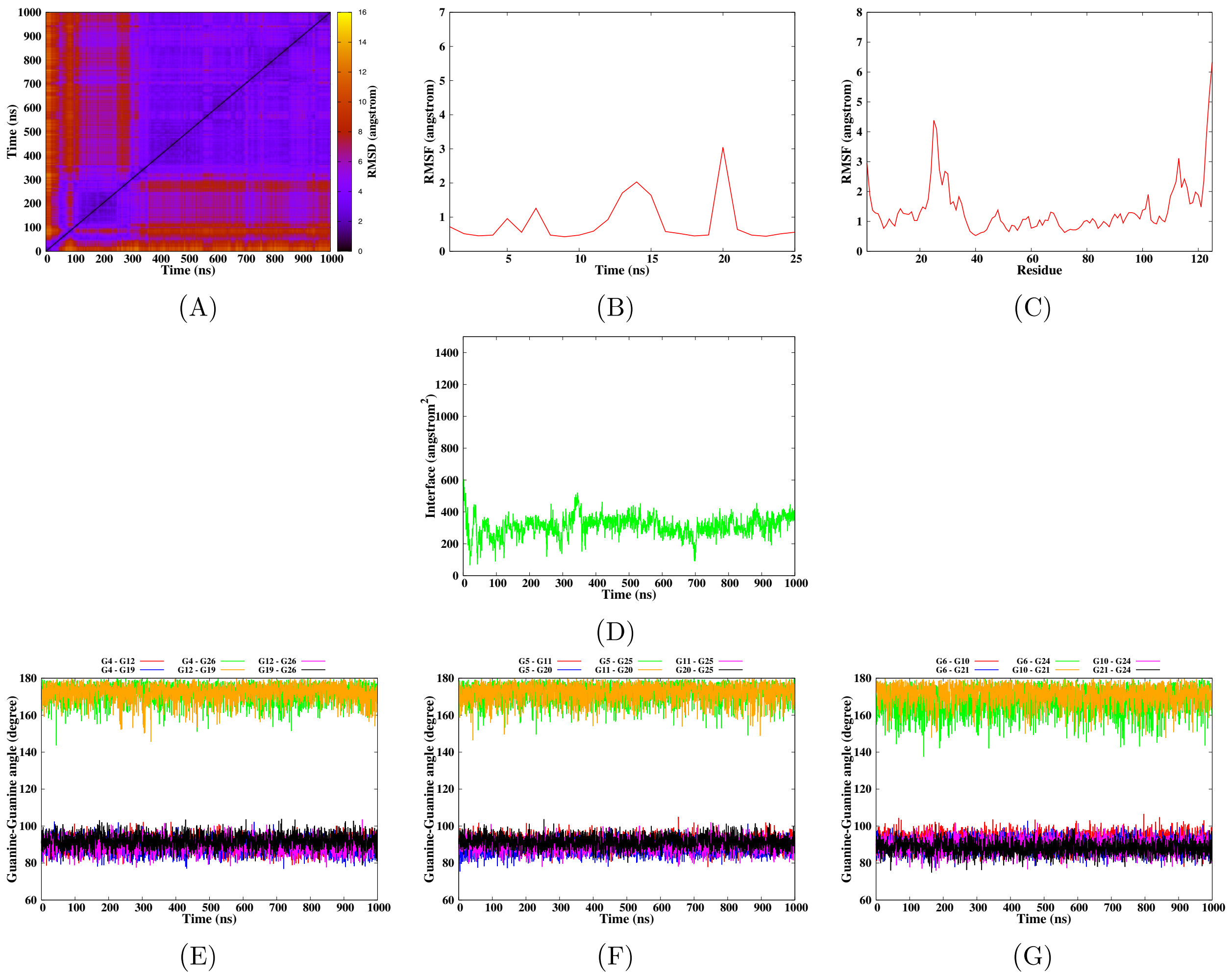

Figure S16 – Simulation of the Bcl-2 G-quadruplex DNA in interaction with 2E4 according to the model 5-1, run 2. The convergence of the simulation is given by the RMSD-2D map of the DNA-Protein complex (A). The mobility of the DNA and protein residues is given by their root mean square fluctuation (B-C). Surface of the interaction interface between the protein and G-quadruplex (D). Finally the structural parameters of the G-quadruplex are given by the angles between the guanines for each tetrad (E-G).

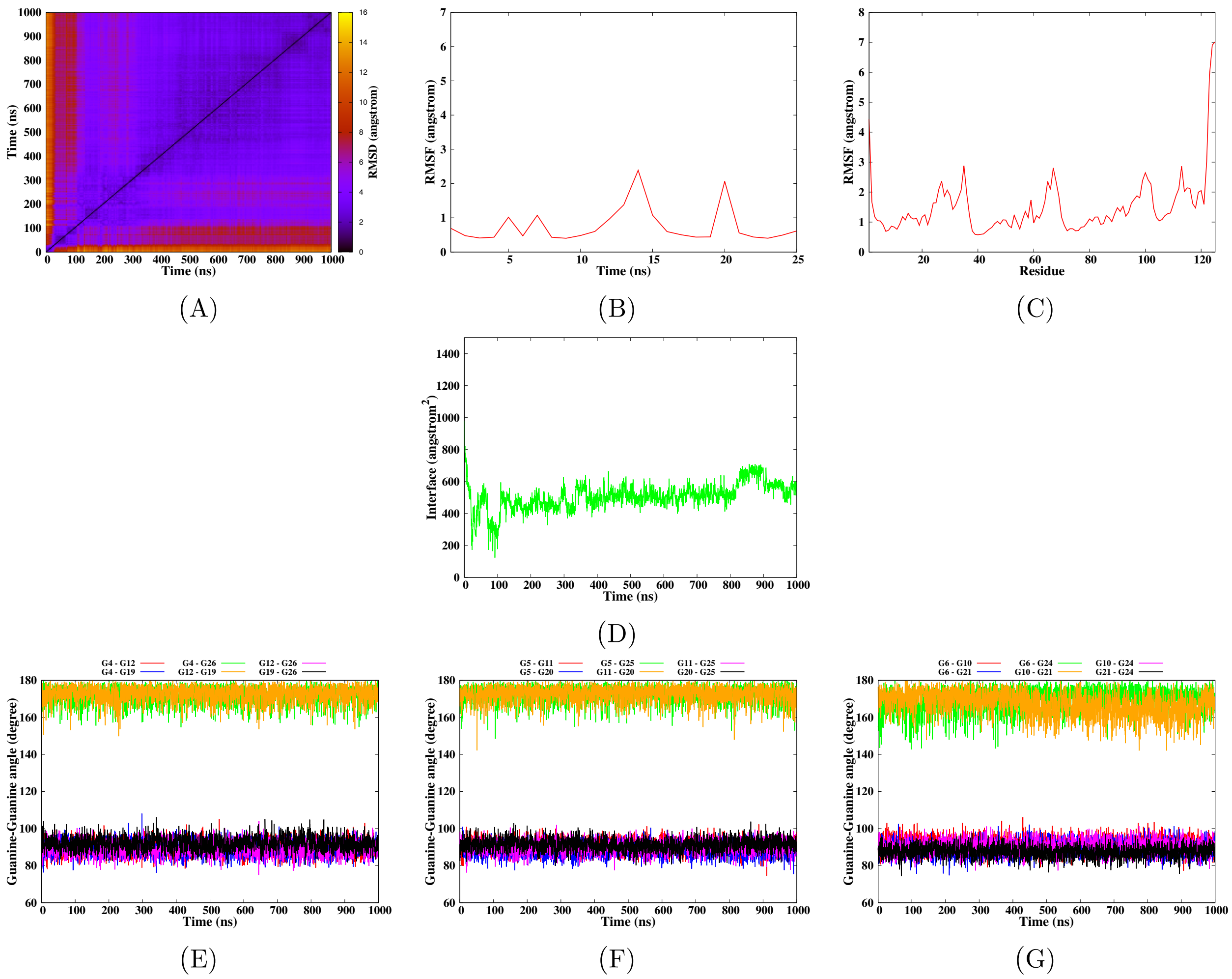

Figure S17 – Simulation of the Bcl-2 G-quadruplex DNA in interaction with 2E4 according to the model 7-1, run 1. The convergence of the simulation is given by the RMSD-2D map of the DNA-Protein complex (A). The mobility of the DNA and protein residues is given by their root mean square fluctuation (B-C). Surface of the interaction interface between the protein and G-quadruplex (D). Finally the structural parameters of the G-quadruplex are given by the angles between the guanines for each tetrad (E-G).

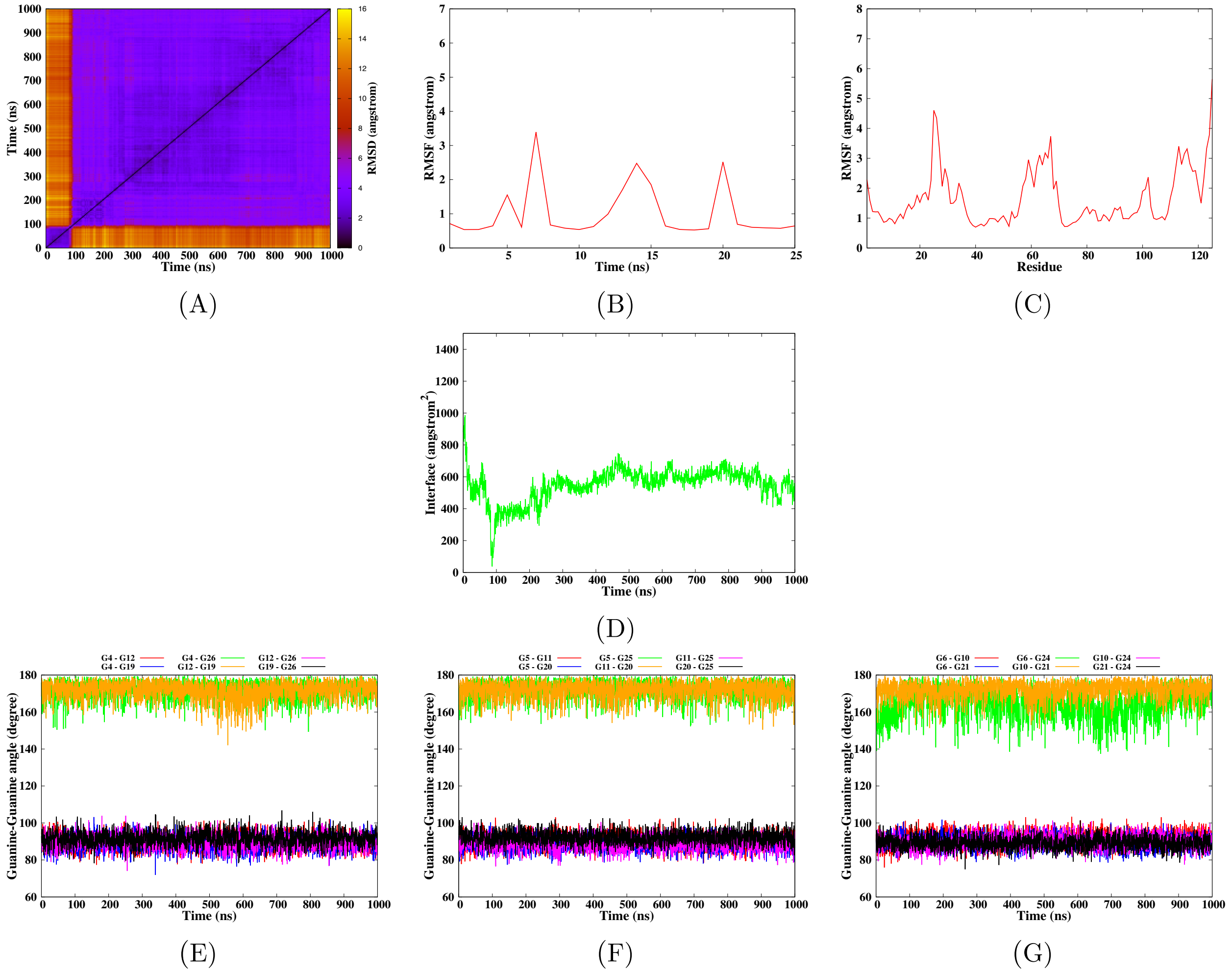

Figure S18 – Simulation of the Bcl-2 G-quadruplex DNA in interaction with 2E4 according to the model 7-1, run 2. The convergence of the simulation is given by the RMSD-2D map of the DNA-Protein complex (A). The mobility of the DNA and protein residues is given by their root mean square fluctuation (B-C). Surface of the interaction interface between the protein and G-quadruplex (D). Finally the structural parameters of the G-quadruplex are given by the angles between the guanines for each tetrad (E-G).

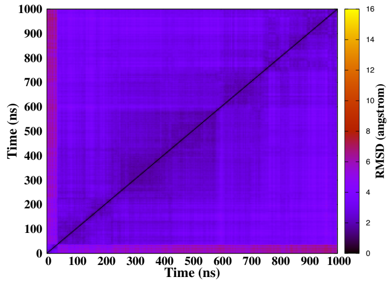

(A)

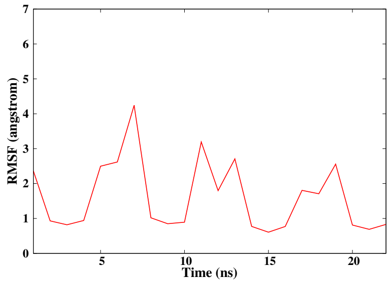

(B)

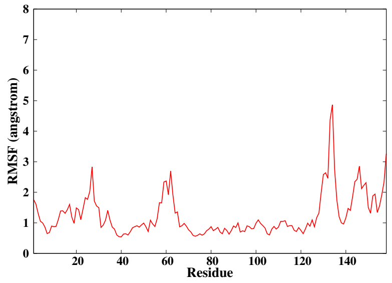

(C)

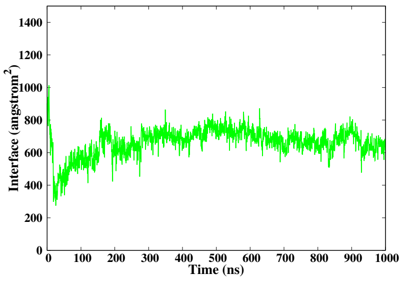

(D)

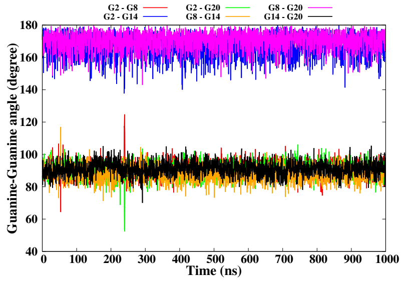

(E)

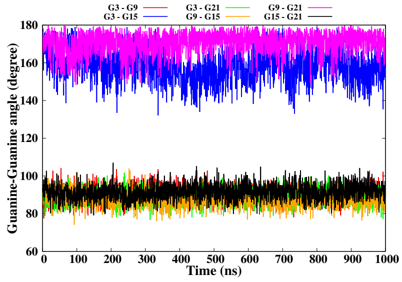

(F)

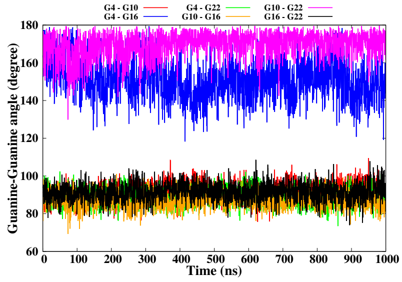

(G)

Figure S19 – Simulation of the h-Telo G-quadruplex DNA in interaction with 2G10 according to the model 13-1, run 1. The convergence of the simulation is given by the RMSD-2D map of the DNA-Protein complex (A). The mobility of the DNA and protein residues is given by their root mean square fluctuation (B-C). Surface of the interaction interface between the protein and G-quadruplex (D). Finally the structural parameters of the G-quadruplex are given by the angles between the guanines for each tetrad (E-G).

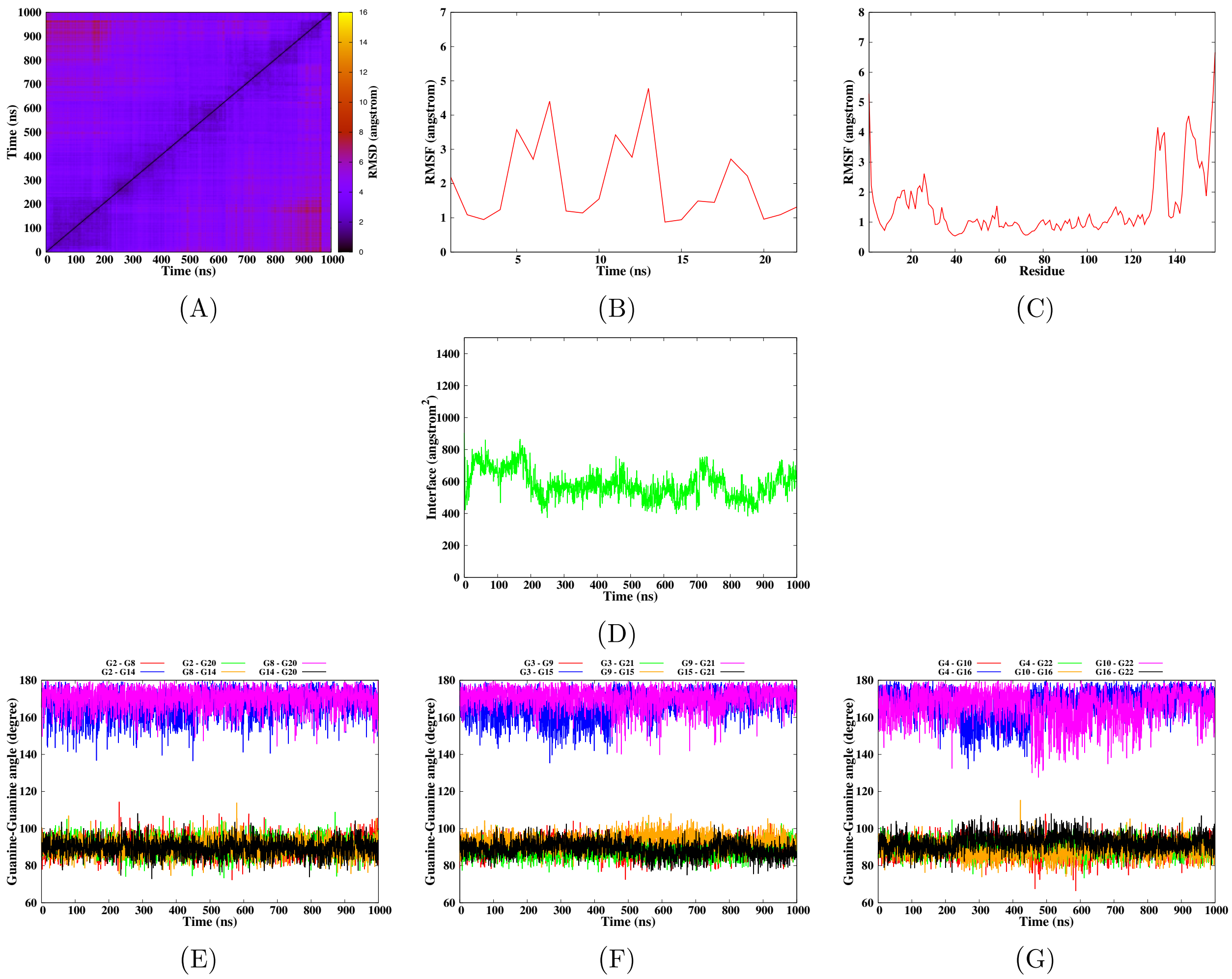

Figure S20 – Simulation of the h-Telo G-quadruplex DNA in interaction with 2G10 according to the model 13-1, run 2. The convergence of the simulation is given by the RMSD-2D map of the DNA-Protein complex (A). The mobility of the DNA and protein residues is given by their root mean square fluctuation (B-C). Surface of the interaction interface between the protein and G-quadruplex (D). Finally the structural parameters of the G-quadruplex are given by the angles between the guanines for each tetrad (E-G).

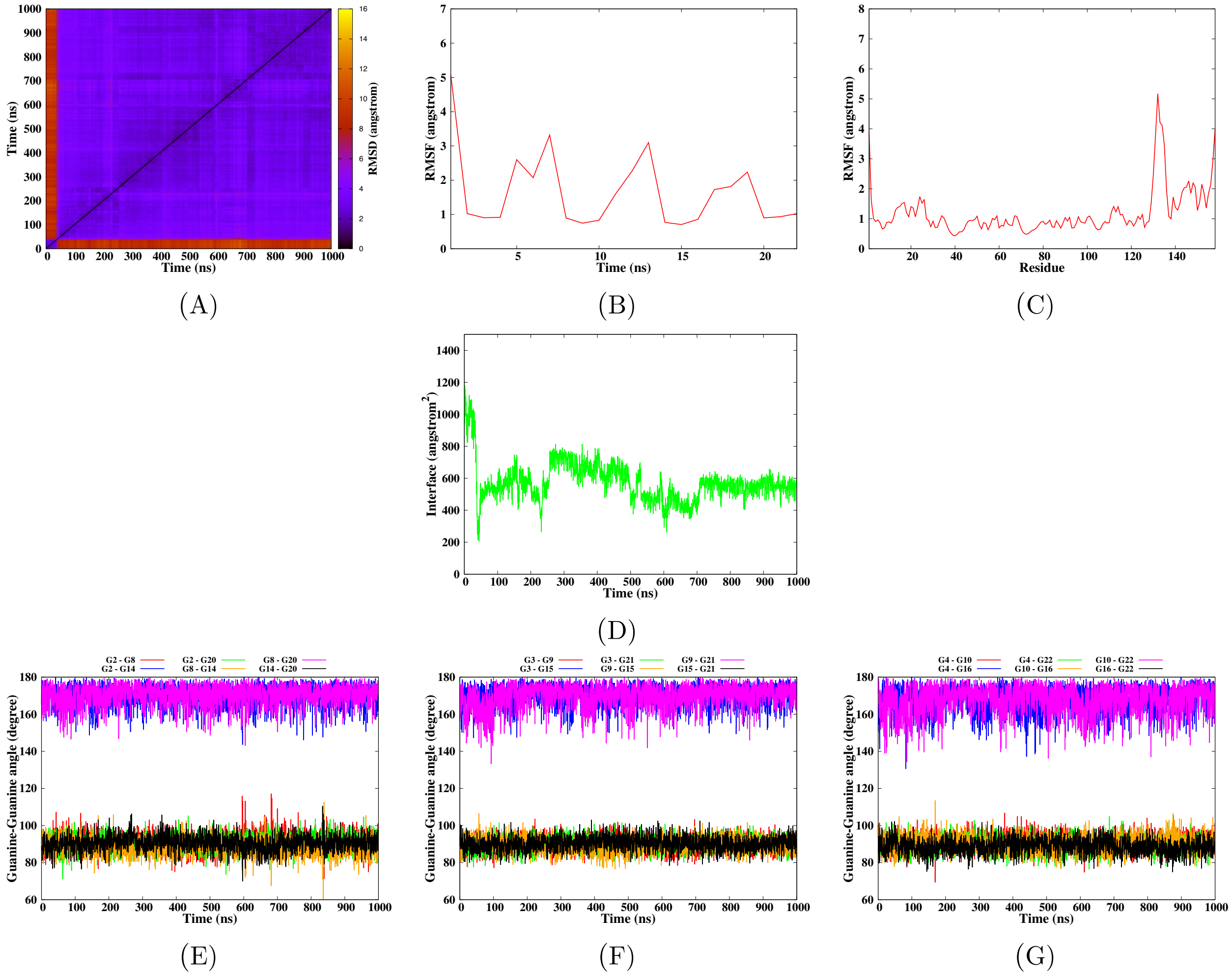

Figure S21 – Simulation of the h-Telo G-quadruplex DNA in interaction with 2G10 according to the model 5-1, run 1. The convergence of the simulation is given by the RMSD-2D map of the DNA-Protein complex (A). The mobility of the DNA and protein residues is given by their root mean square fluctuation (B-C). Surface of the interaction interface between the protein and G-quadruplex (D). Finally the structural parameters of the G-quadruplex are given by the angles between the guanines for each tetrad (E-G).

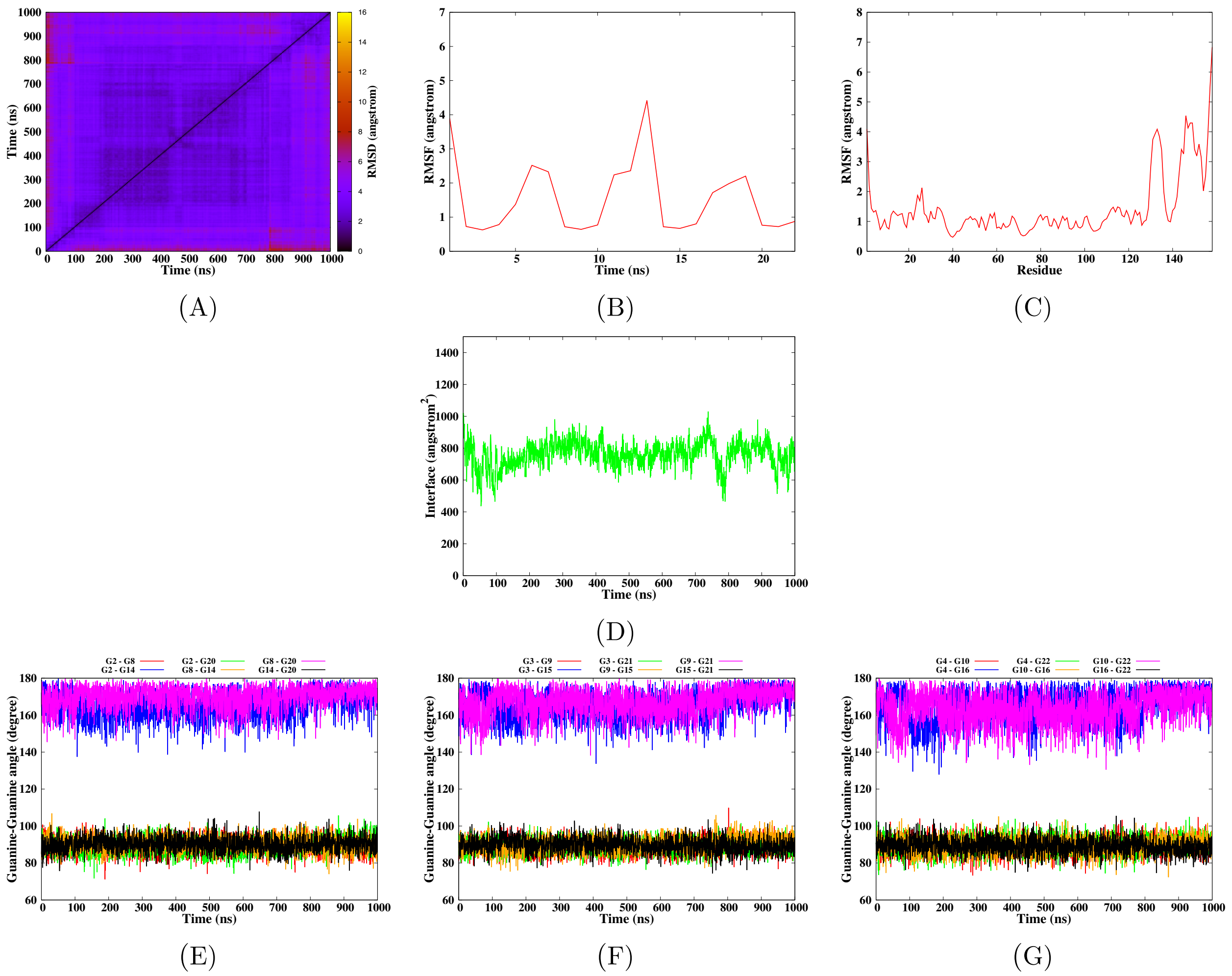

Figure S22 – Simulation of the h-Telo G-quadruplex DNA in interaction with 2G10 according to the model 5-1, run 2. The convergence of the simulation is given by the RMSD-2D map of the DNA-Protein complex (A). The mobility of the DNA and protein residues is given by their root mean square fluctuation (B-C). Surface of the interaction interface between the protein and G-quadruplex (D). Finally the structural parameters of the G-quadruplex are given by the angles between the guanines for each tetrad (E-G).

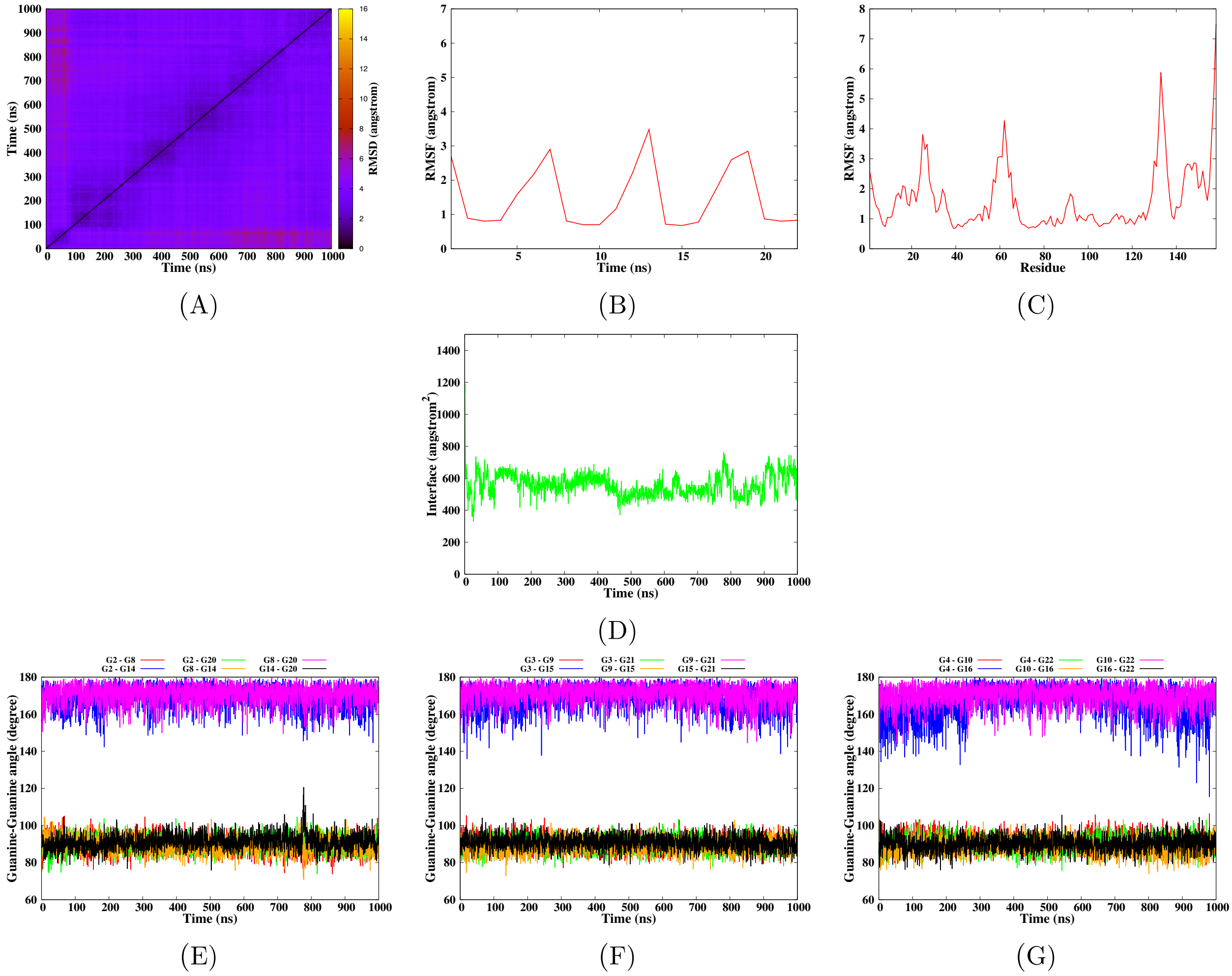

Figure S23 – Simulation of the h-Telo G-quadruplex DNA in interaction with 2G10 according to the model 6-1, run 1. The convergence of the simulation is given by the RMSD-2D map of the DNA-Protein complex (A). The mobility of the DNA and protein residues is given by their root mean square fluctuation (B-C). Surface of the interaction interface between the protein and G-quadruplex (D). Finally the structural parameters of the G-quadruplex are given by the angles between the guanines for each tetrad (E-G).

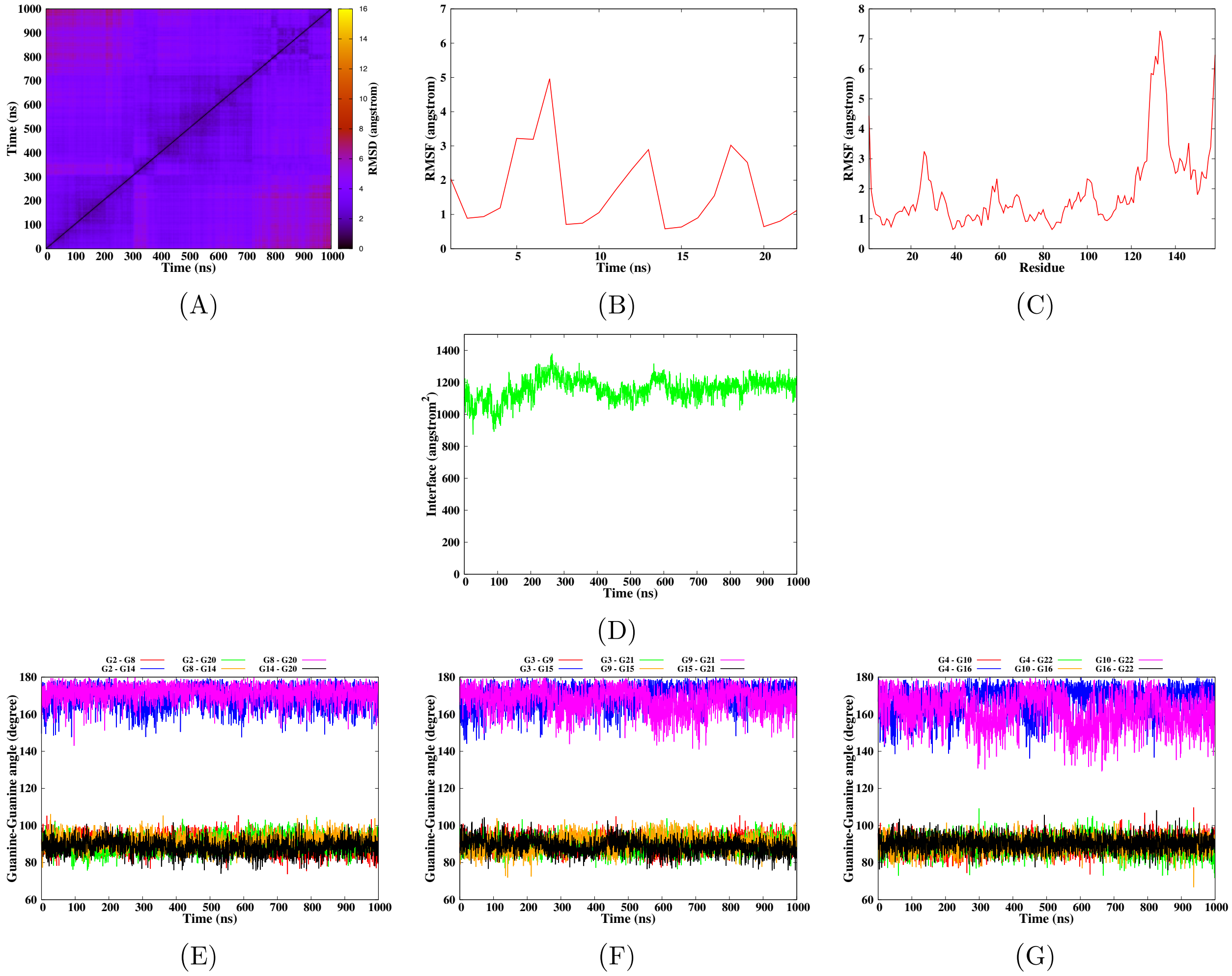

Figure S24 – Simulation of the h-Telo G-quadruplex DNA in interaction with 2G10 according to the model 6-1, run 2. The convergence of the simulation is given by the RMSD-2D map of the DNA-Protein complex (A). The mobility of the DNA and protein residues is given by their root mean square fluctuation (B-C). Surface of the interaction interface between the protein and G-quadruplex (D). Finally the structural parameters of the G-quadruplex are given by the angles between the guanines for each tetrad (E-G).

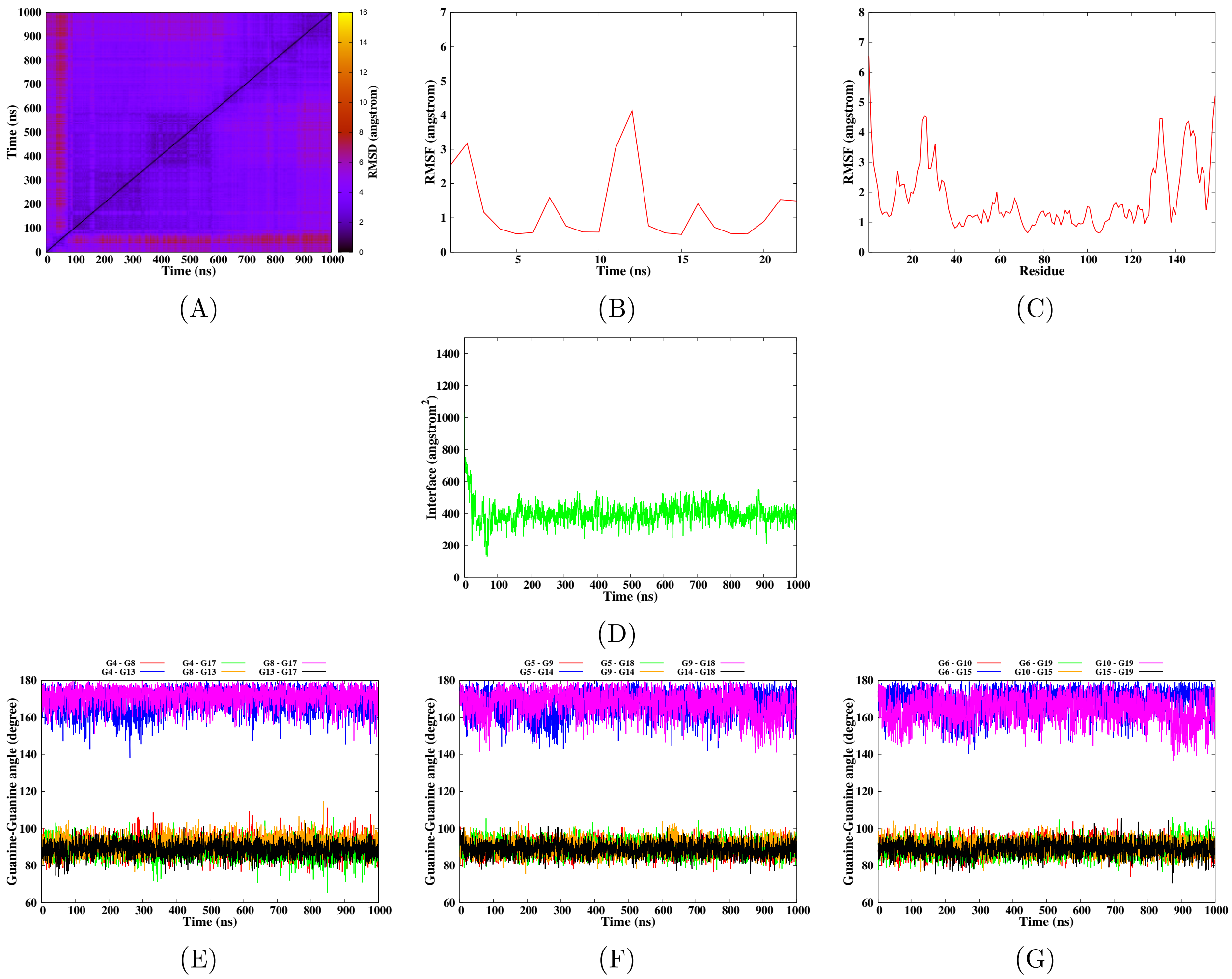

Figure S25 – Simulation of the c-Myc G-quadruplex DNA in interaction with 2G10 according to the model 1-1, run 1. The convergence of the simulation is given by the RMSD-2D map of the DNA-Protein complex (A). The mobility of the DNA and protein residues is given by their root mean square fluctuation (B-C). Surface of the interaction interface between the protein and G-quadruplex (D). Finally the structural parameters of the G-quadruplex are given by the angles between the guanines for each tetrad (E-G).

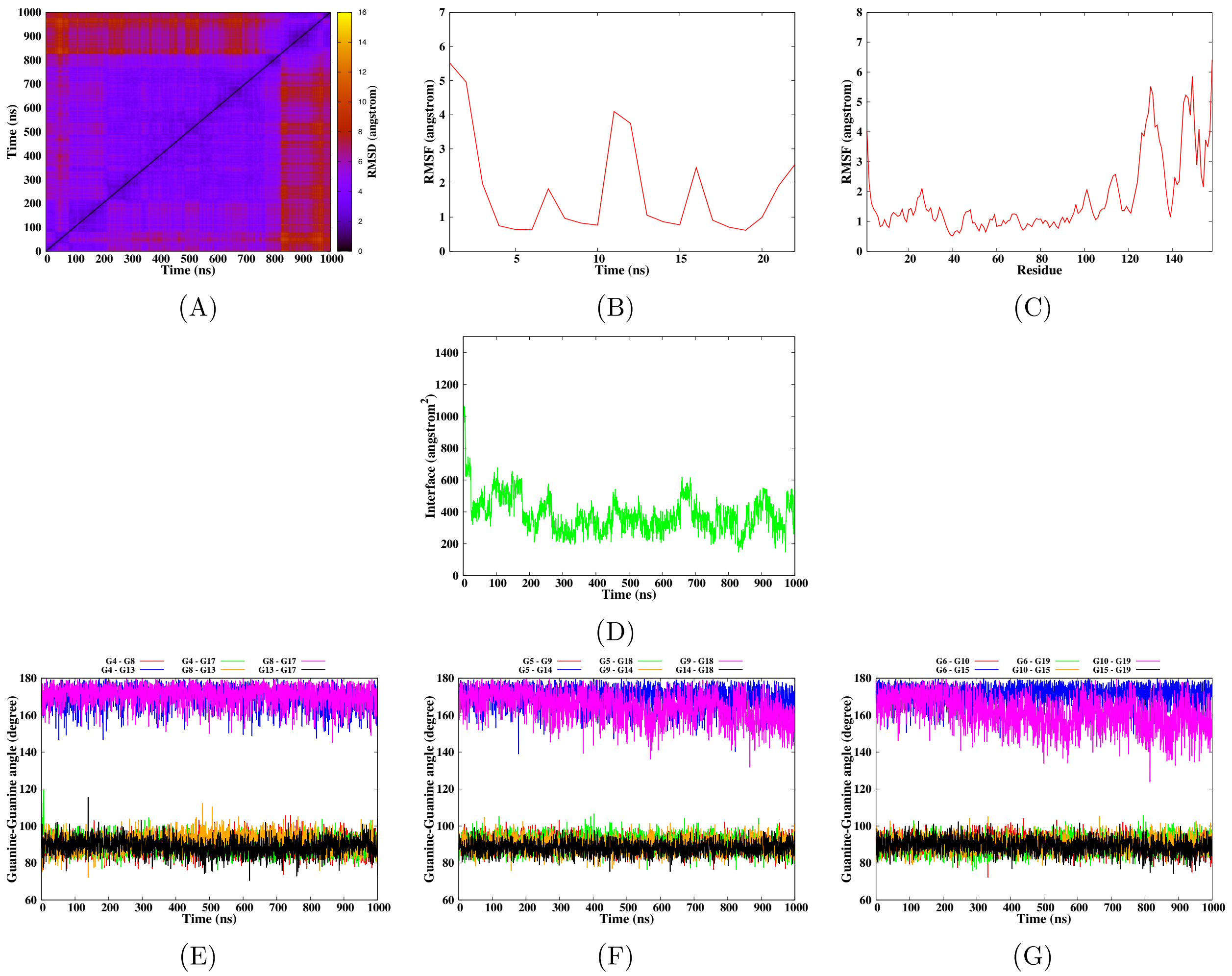

Figure S26 – Simulation of the c-Myc G-quadruplex DNA in interaction with 2G10 according to the model 1-1, run 2. The convergence of the simulation is given by the RMSD-2D map of the DNA-Protein complex (A). The mobility of the DNA and protein residues is given by their root mean square fluctuation (B-C). Surface of the interaction interface between the protein and G-quadruplex (D). Finally the structural parameters of the G-quadruplex are given by the angles between the guanines for each tetrad (E-G).

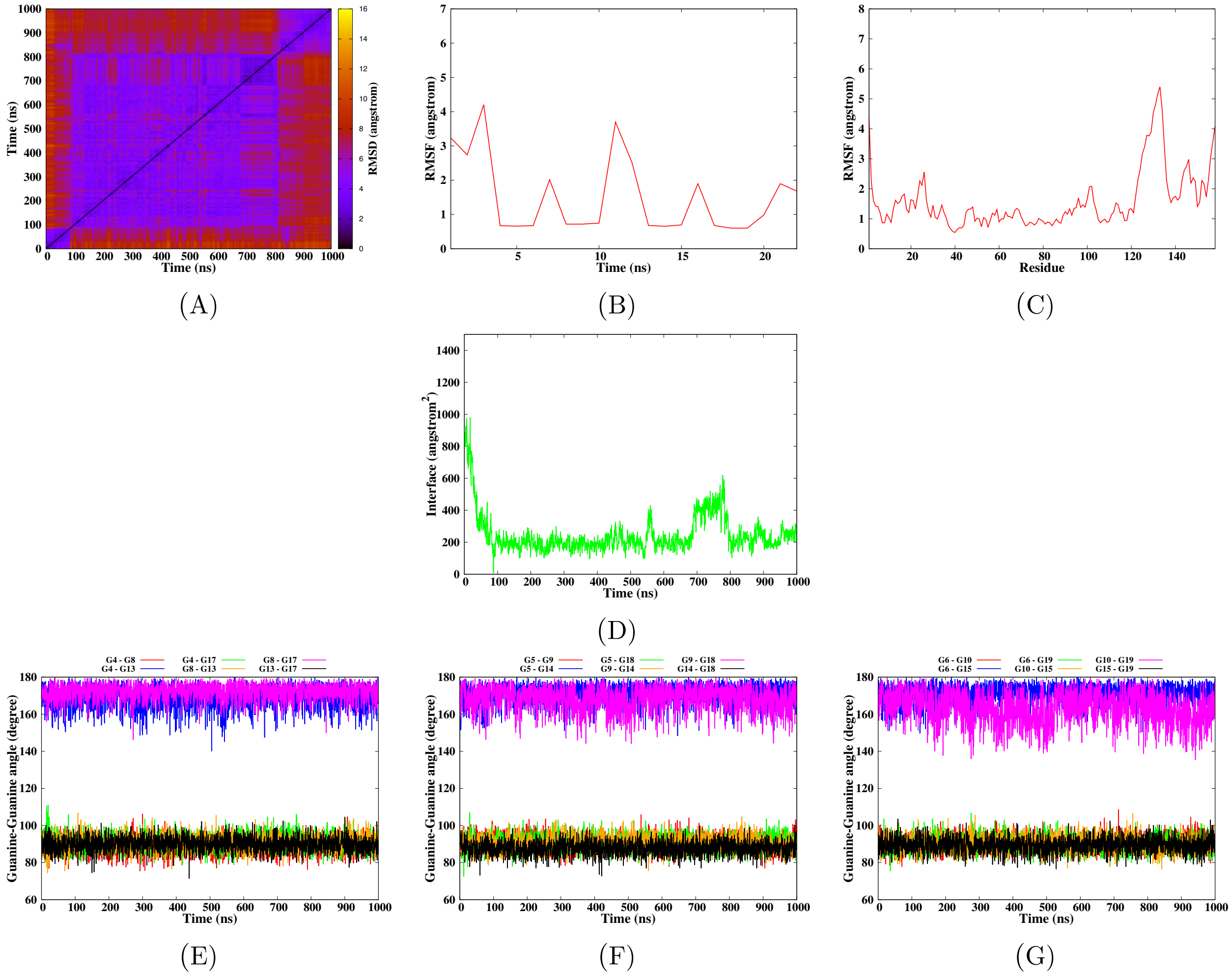

Figure S27 – Simulation of the c-Myc G-quadruplex DNA in interaction with 2G10 according to the model 2-1, run 1. The convergence of the simulation is given by the RMSD-2D map of the DNA-Protein complex (A). The mobility of the DNA and protein residues is given by their root mean square fluctuation (B-C). Surface of the interaction interface between the protein and G-quadruplex (D). Finally the structural parameters of the G-quadruplex are given by the angles between the guanines for each tetrad (E-G).

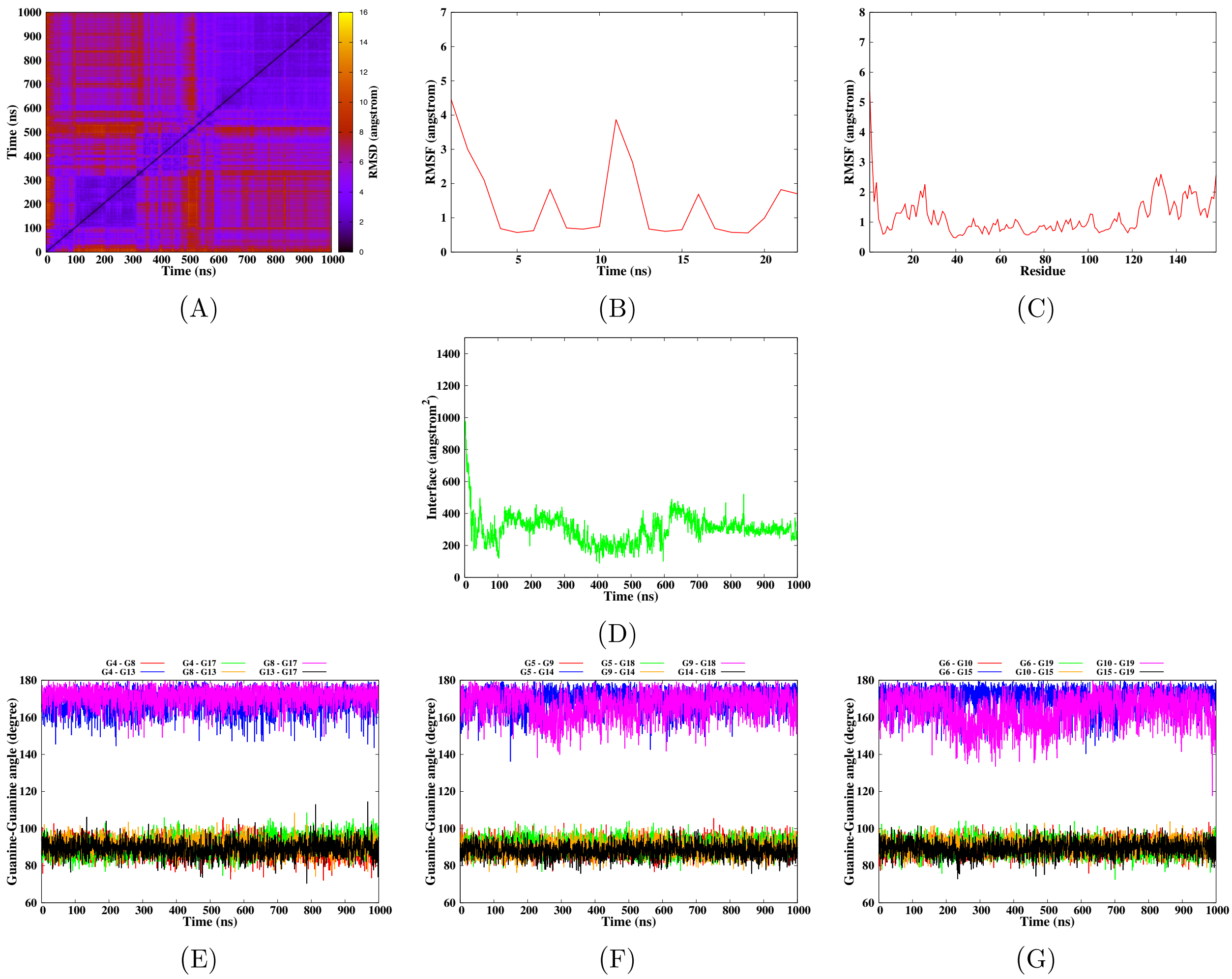

Figure S28 – Simulation of the c-Myc G-quadruplex DNA in interaction with 2G10 according to the model 2-1, run 2. The convergence of the simulation is given by the RMSD-2D map of the DNA-Protein complex (A). The mobility of the DNA and protein residues is given by their root mean square fluctuation (B-C). Surface of the interaction interface between the protein and G-quadruplex (D). Finally the structural parameters of the G-quadruplex are given by the angles between the guanines for each tetrad (E-G).

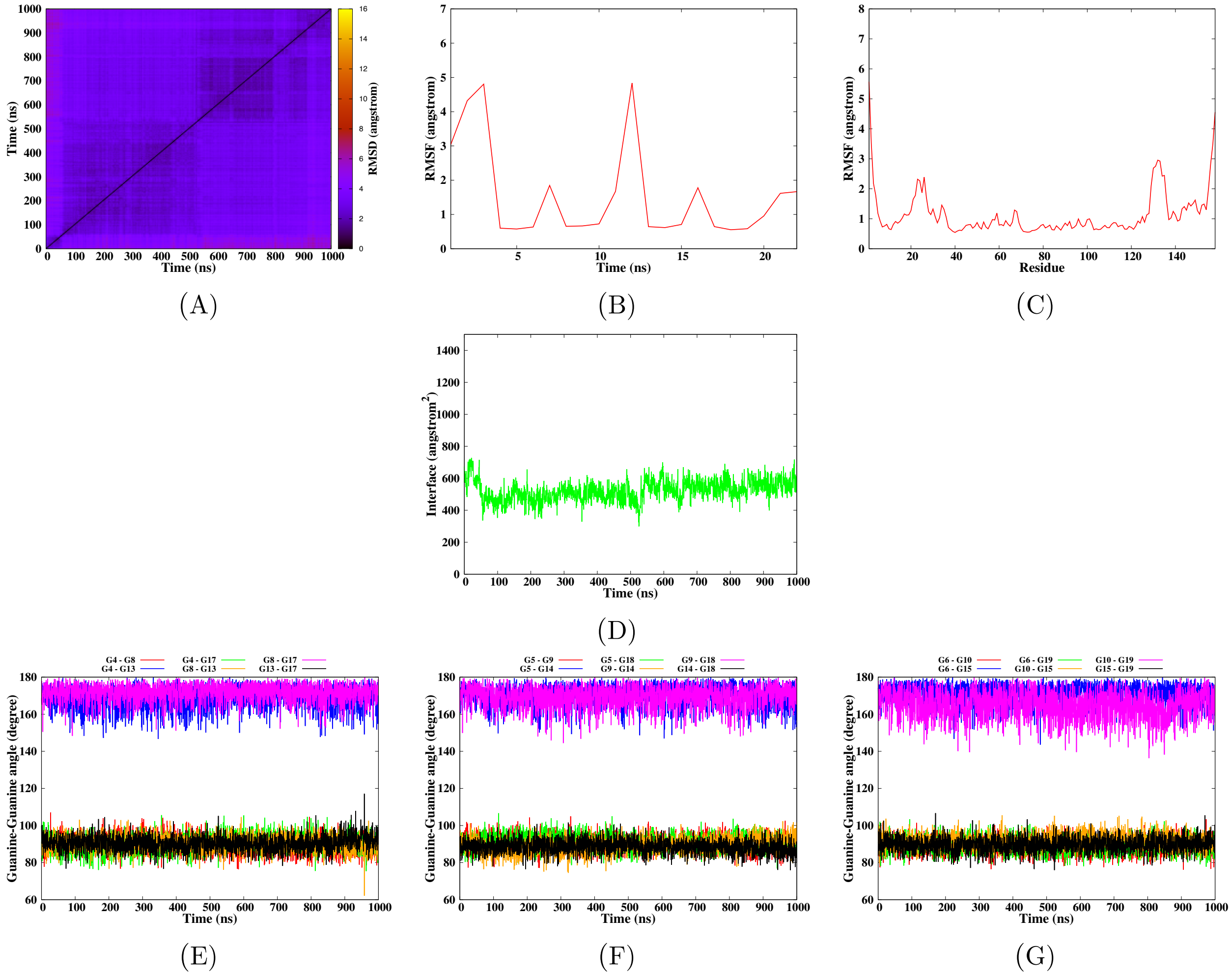

Figure S29 – Simulation of the c-Myc G-quadruplex DNA in interaction with 2G10 according to the model 3-1, run 1. The convergence of the simulation is given by the RMSD-2D map of the DNA-Protein complex (A). The mobility of the DNA and protein residues is given by their root mean square fluctuation (B-C). Surface of the interaction interface between the protein and G-quadruplex (D). Finally the structural parameters of the G-quadruplex are given by the angles between the guanines for each tetrad (E-G).

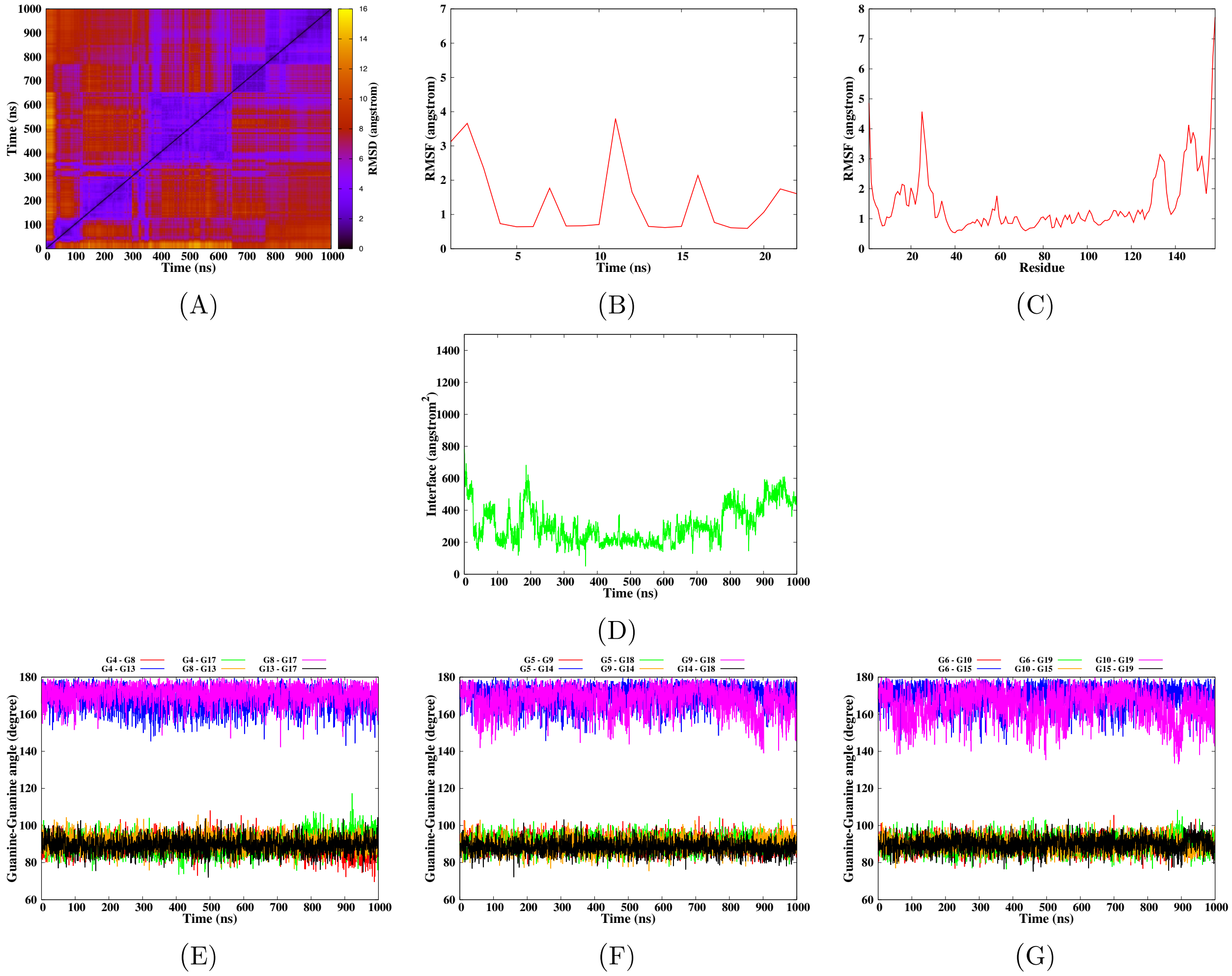

Figure S30 – Simulation of the c-Myc G-quadruplex DNA in interaction with 2G10 according to the model 3-1, run 2. The convergence of the simulation is given by the RMSD-2D map of the DNA-Protein complex (A). The mobility of the DNA and protein residues is given by their root mean square fluctuation (B-C). Surface of the interaction interface between the protein and G-quadruplex (D). Finally the structural parameters of the G-quadruplex are given by the angles between the guanines for each tetrad (E-G).

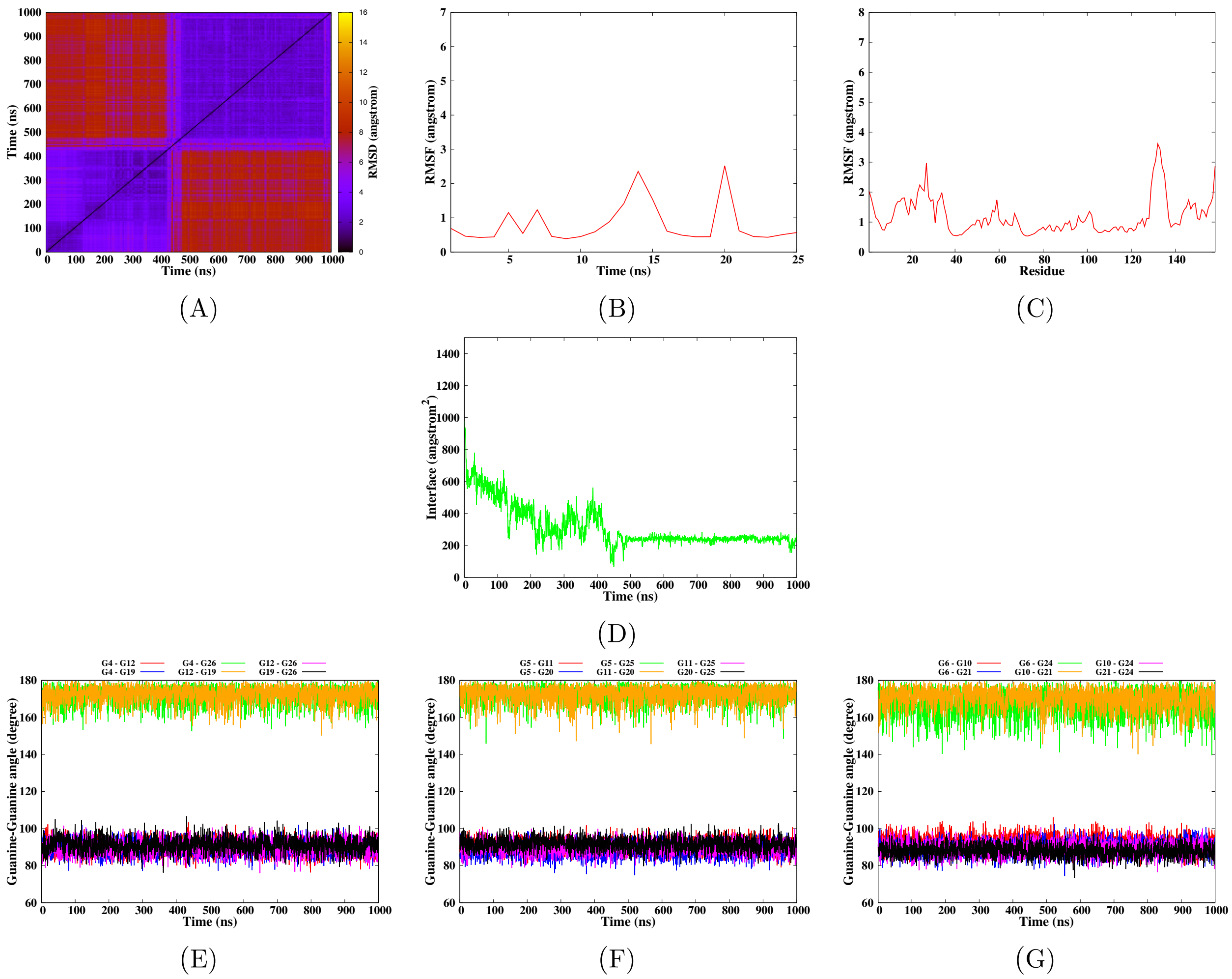

Figure S31 – Simulation of the Bcl-2 G-quadruplex DNA in interaction with 2G10 according to the model 1-1, run 1. The convergence of the simulation is given by the RMSD-2D map of the DNA-Protein complex (A). The mobility of the DNA and protein residues is given by their root mean square fluctuation (B-C). Surface of the interaction interface between the protein and G-quadruplex (D). Finally the structural parameters of the G-quadruplex are given by the angles between the guanines for each tetrad (E-G).

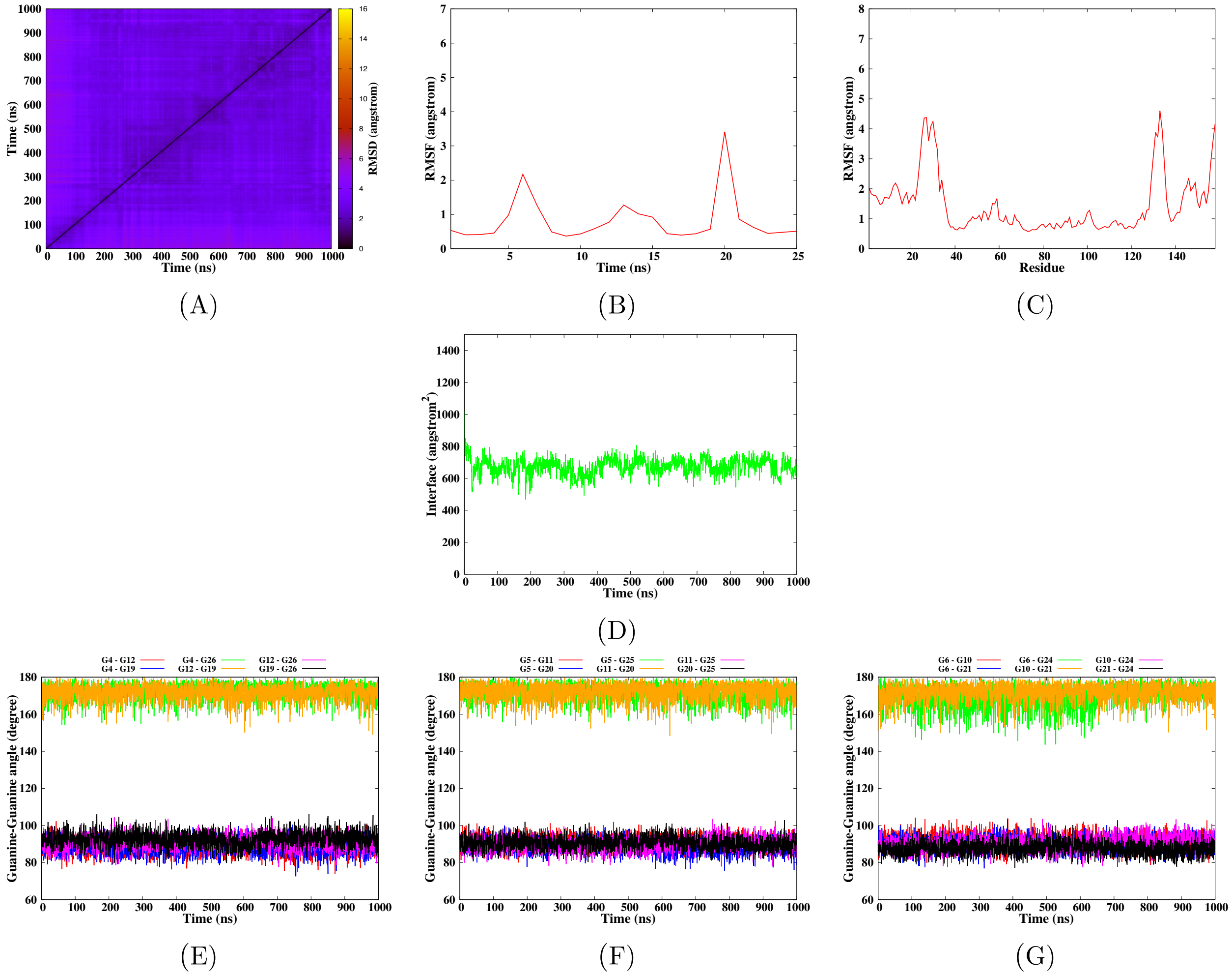

Figure S32 – Simulation of the Bcl-2 G-quadruplex DNA in interaction with 2G10 according to the model 1-1, run 2. The convergence of the simulation is given by the RMSD-2D map of the DNA-Protein complex (A). The mobility of the DNA and protein residues is given by their root mean square fluctuation (B-C). Surface of the interaction interface between the protein and G-quadruplex (D). Finally the structural parameters of the G-quadruplex are given by the angles between the guanines for each tetrad (E-G).

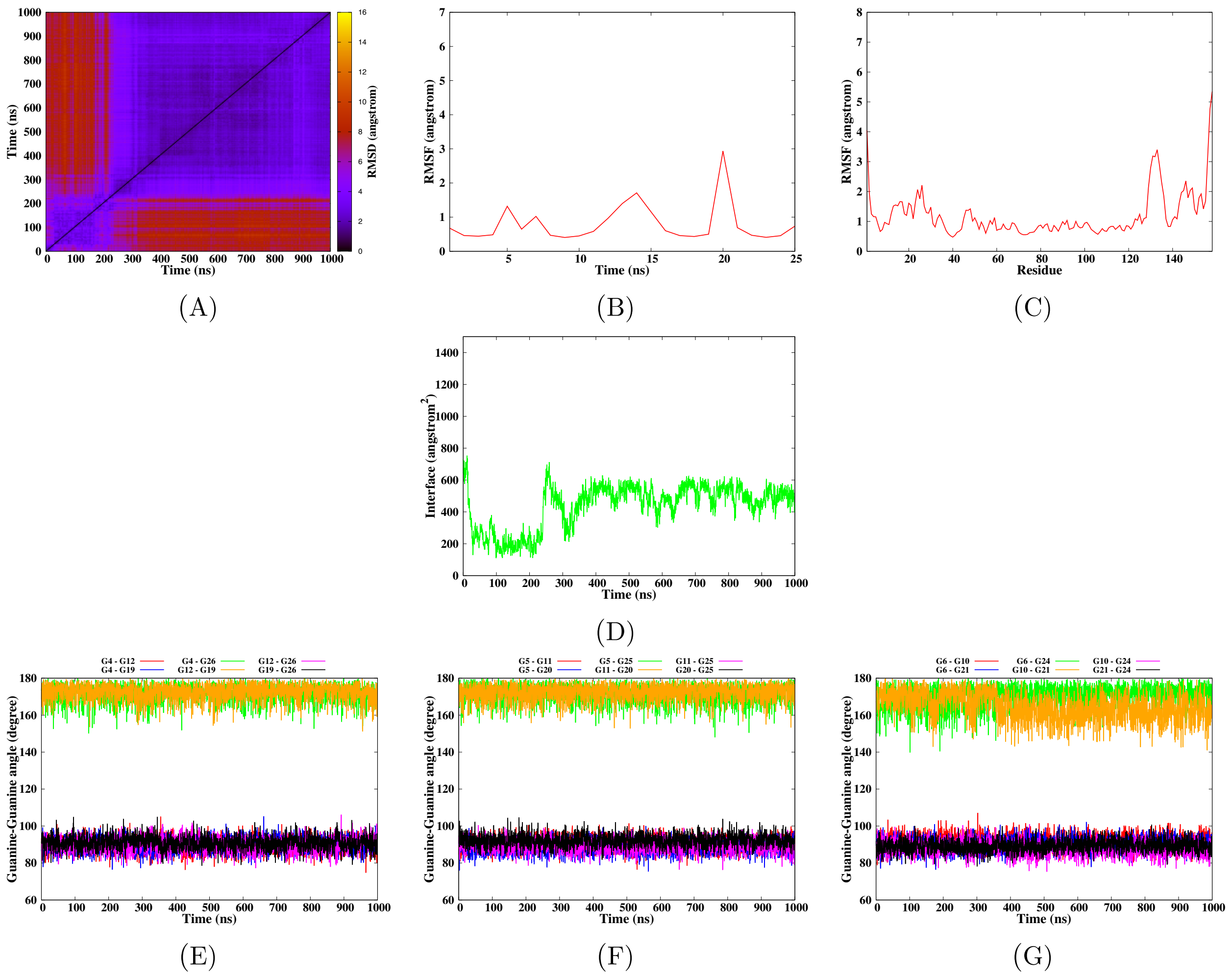

Figure S33 – Simulation of the Bcl-2 G-quadruplex DNA in interaction with 2G10 according to the model 13-1, run 1. The convergence of the simulation is given by the RMSD-2D map of the DNA-Protein complex (A). The mobility of the DNA and protein residues is given by their root mean square fluctuation (B-C). Surface of the interaction interface between the protein and G-quadruplex (D). Finally the structural parameters of the G-quadruplex are given by the angles between the guanines for each tetrad (E-G).

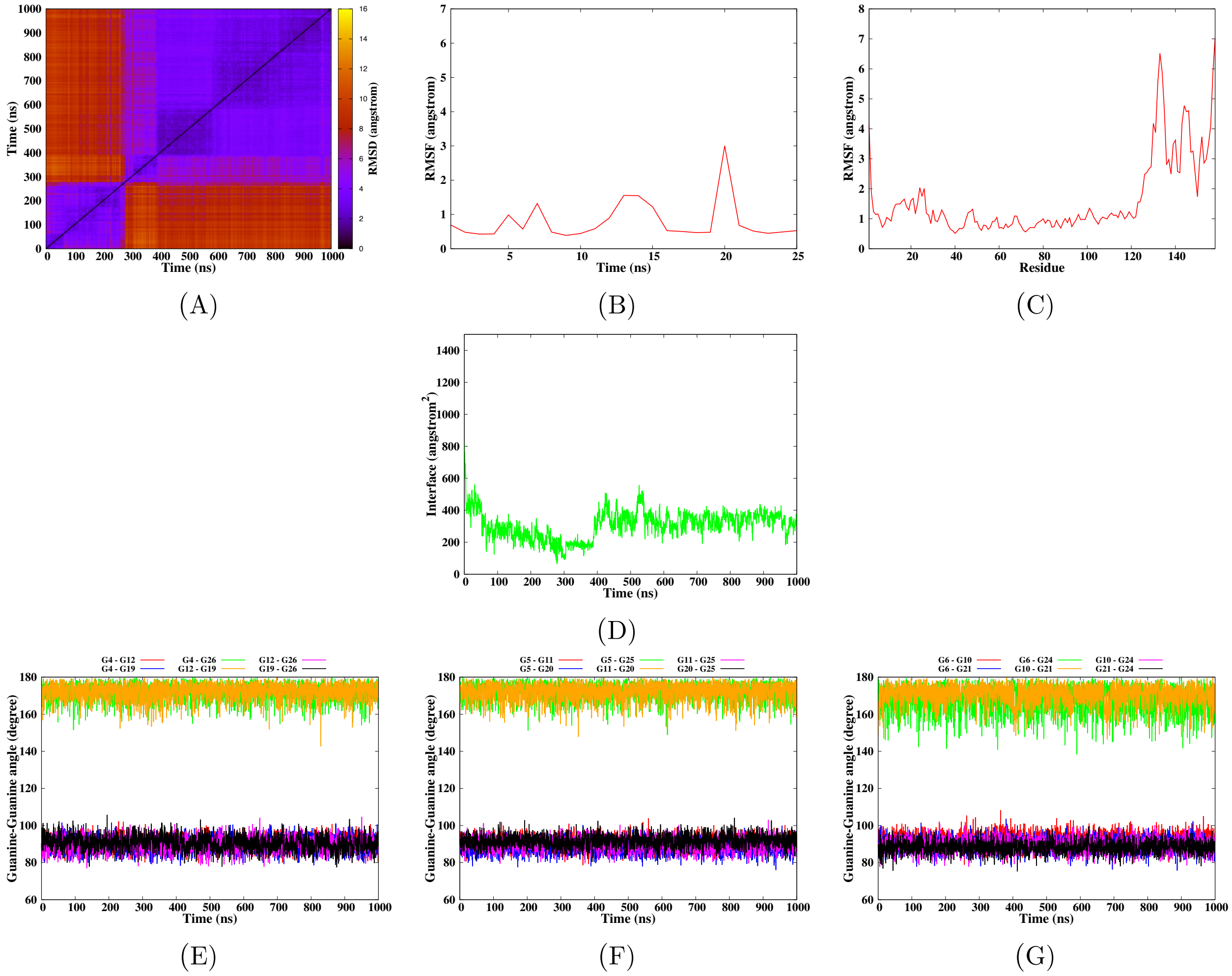

Figure S34 – Simulation of the Bcl-2 G-quadruplex DNA in interaction with 2G10 according to the model 13-1, run 2. The convergence of the simulation is given by the RMSD-2D map of the DNA-Protein complex (A). The mobility of the DNA and protein residues is given by their root mean square fluctuation (B-C). Surface of the interaction interface between the protein and G-quadruplex (D). Finally the structural parameters of the G-quadruplex are given by the angles between the guanines for each tetrad (E-G).

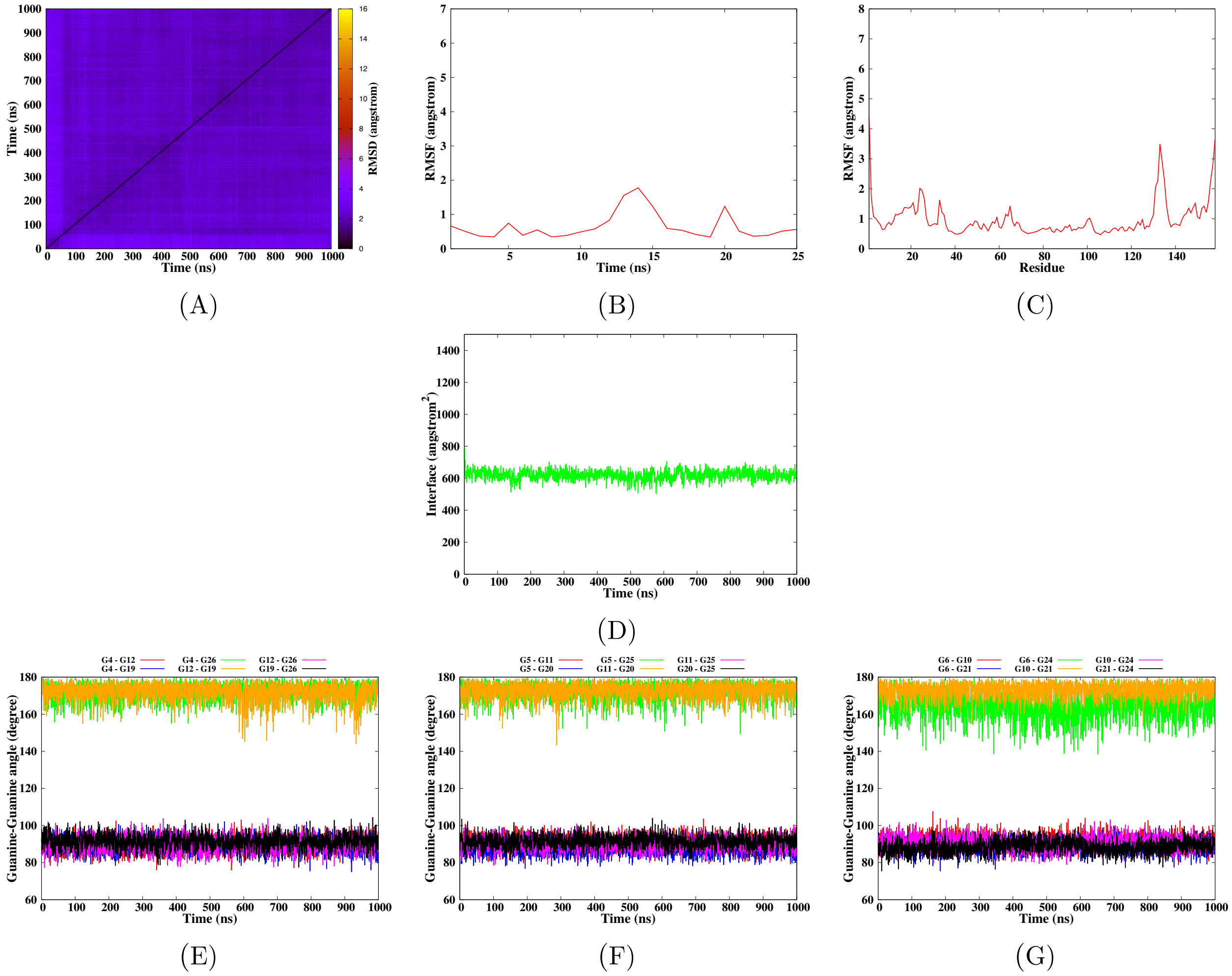

Figure S35 – Simulation of the Bcl-2 G-quadruplex DNA in interaction with 2G10 according to the model 3-1, run 1. The convergence of the simulation is given by the RMSD-2D map of the DNA-Protein complex (A). The mobility of the DNA and protein residues is given by their root mean square fluctuation (B-C). Surface of the interaction interface between the protein and G-quadruplex (D). Finally the structural parameters of the G-quadruplex are given by the angles between the guanines for each tetrad (E-G).

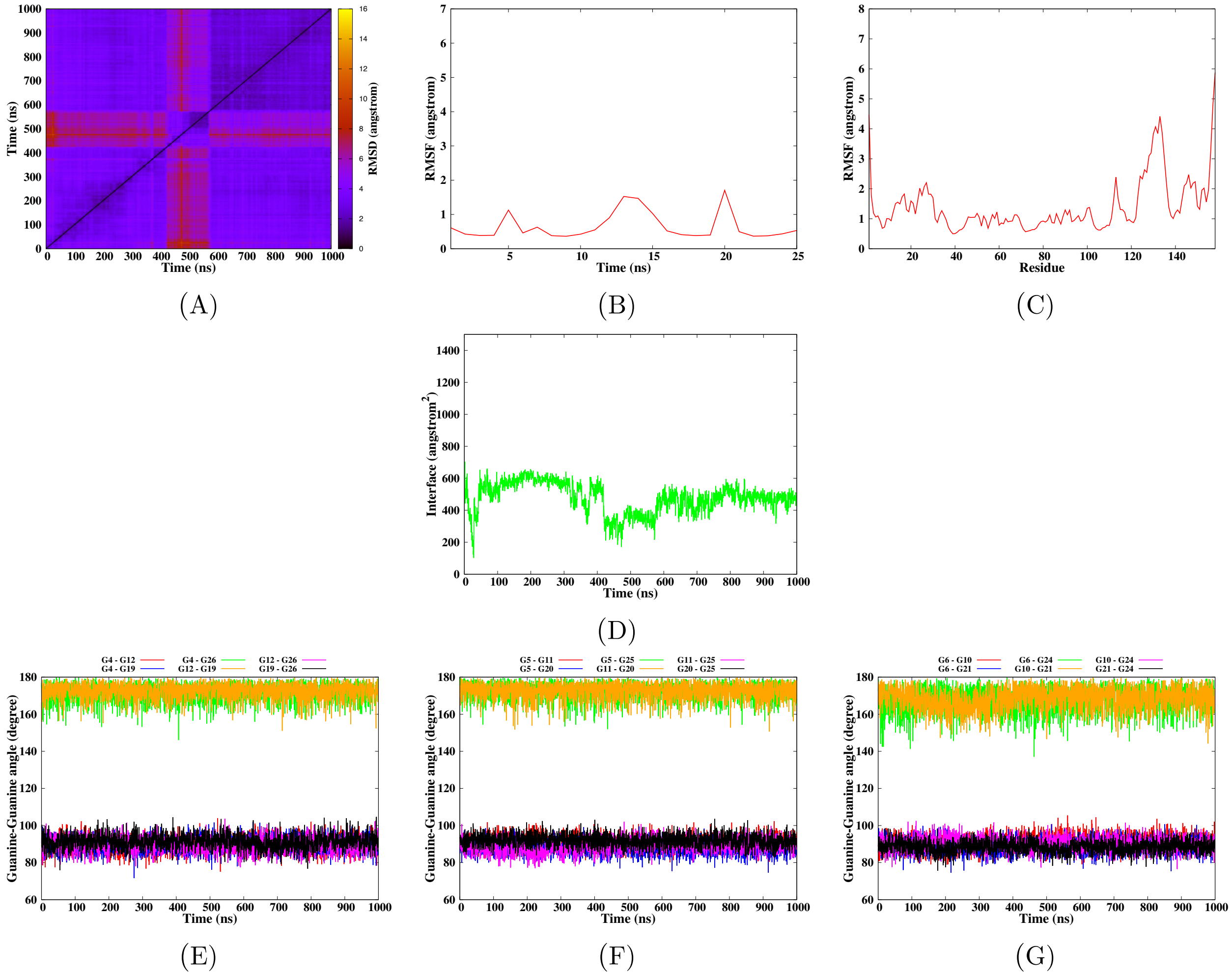

Figure S36 – Simulation of the Bcl-2 G-quadruplex DNA in interaction with 2G10 according to the model 3-1, run 2. The convergence of the simulation is given by the RMSD-2D map of the DNA-Protein complex (A). The mobility of the DNA and protein residues is given by their root mean square fluctuation (B-C). Surface of the interaction interface between the protein and G-quadruplex (D). Finally the structural parameters of the G-quadruplex are given by the angles between the guanines for each tetrad (E-G).

A)

```
        1        10        20        30        40
2E4     SDLGKKLLEAARAGQDDEVRILMANGADVNAKDRYGNTPLHLAAY
DM102   ---------------------------------------------

        46  50        60        70        80        90
2E4     MGHLEIVEVLLKYGADVNAMDHWGRTPLHLAASRGHLEIVEVLLK
DM102   PGHLKGRRIGLWYASKQ-----------------GQK--------
        ***:  .: * *.:..                  *:

        91       100       110       120 124
2E4     HGADVNAQDKFGKTAFDISIDNGNEDLAEILQKLN
DM102   --------NK-------------------------
                :*
```

B)

```
        1        10        20        30        40
2E4     SDLGKKLLEAARAGQDDEVRILMANGADVNAKDRYGNTPLHLAAY
DM102   --------------------------------------P------
                                              *

        46  50        60        70        80        90
2E4     MGHLEIVEVLLKYGADVNAMDHWGRTPLHLAASRGHLEIVEVLLK
DM102   -GHLKGR-----------------RIGLWYASKQ-----------
         ***:                   *  *  *:.:

        91       100       110       120 124
2E4     HGADVNAQDKFGKTAFDISIDNGNEDLAEILQKLN
DM102   ------------------------------GQKNK
                                       ** :
```

Figure S37 - Alignment of 2E4 DARPin with the *c-Myc* specific DM102 peptide using A) Clusta Omega and B) M-Coffee. Amino acids mutated by Scholz et al. [25] and not conserved in all DARPins are represented in red. In black bold, the amino acids P72 is replaced by an S in 2G10.

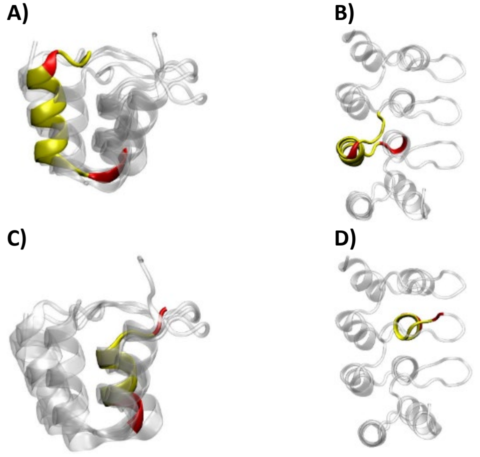

Figure S38 - Area of the protein aligned to the peptide (yellow) with Clustal Omega in A) side view and B) top view, with the location of the modifications made by Scholz et al. in red. C) and D) check the same representations for the alignment with M-Coffee.

Clustal Omega

```
2G10    SDLGKKLLEAARAGQDDEVRILMANGADVNAIDNIGQTPLHLAAAWGHLEIVEVLLKHGADVNAMDRWGRTSLHLAAKWGHLEIVEVLLKHGADVNARDKKGFTPLHLAAIWGHLEIVEVLLKHGADVNAQDKFGKTAFDISIDNGNEDLAEILQKLN
DM102   --------------------------------------PGHLK-------GR-----------RIGLWY------ASKQGQKN----------------K----------------------------------------------------------
                                              * **                      :. *       *:* *: :                *
```

M-Coffee

```
2G10    SDLGKKLLEAARAGQDDEVRILMANGADVNAIDNIGQTPLHLAAAWGHLEIVEVLLKHGADVNAMDRWGRTSLHLAAKWGHLEIVEVLLKHGADVNARDKKGFTPLHLAAIWGHLEIVEVLLKHGADVNAQDKFGKTAFDISIDNGNEDLAEILQKLN
DM102   --------------------------------------PG-------HLKG-----------------RRIGLWYASK----------------QGQ----------------------------------------------------------KNK
                                              *        **:                   * .*  *:*                 .                                                           * :
```

Figure S39 - Alignment of DM102 peptide with DARPin 2G10 protein using the Clustal Omega method and the M-Coffee algorithm.
